# Vegetable Gardening and Health Outcomes in Older Cancer Survivors: A Randomized Clinical Trial

**DOI:** 10.1001/jamanetworkopen.2024.17122

**Published:** 2024-06-20

**Authors:** Wendy Demark-Wahnefried, Robert A. Oster, Kerry P. Smith, Harleen Kaur, Andrew D. Frugé, W. Walker Cole, Julie L. Locher, Gabrielle B. Rocque, Maria Pisu, Jennifer R. Bail, Harvey Jay Cohen, Douglas R. Moellering, Cindy K. Blair

**Affiliations:** 1 Department of Nutrition Sciences, University of Alabama at Birmingham; 2 Department of Medicine, University of Alabama at Birmingham; 3 Alabama Cooperative Extension System, Auburn University, Auburn; 4 College of Nursing, Auburn University, Auburn, Alabama; 5 Department of Health Behavior, University of Alabama at Birmingham; 6 University of Alabama in Huntsville; 7 Department of Medicine, Duke University Medical Center, Durham, North Carolina; 8 Department of Internal Medicine, University of New Mexico, Albuquerque

## Abstract

**Importance:**

Cancer survivors experience accelerated functional decline that threatens independence and quality of life. Previous studies have suggested that vegetable gardening may improve diet, physical activity, and physical function in this vulnerable population, which comprises more than 5% of the US population.

**Objective:**

To assess whether diet, physical activity and functioning, and other outcomes improved in older cancer survivors assigned to a vegetable gardening intervention compared with a waitlist.

**Design, Setting, and Participants:**

From May 11, 2016, to May 2, 2022, a 2-arm, assessor-blinded, crossover-designed, intent-to-treat, randomized clinical trial was conducted at cancer survivors’ homes across Alabama. Medicare-eligible survivors of cancers with 5-year survival of 60% or more were registry ascertained and screened for suboptimal vegetable and fruit consumption (<5 servings per day), physical activity (<150 moderate-to-vigorous minutes per week), and physical function (36-Item Short Form Health Survey [SF-36] subscale score ≤90). Consented participants underwent baseline assessments, were randomly assigned to intervention or waitlisted arms, and were reassessed at 1-year follow-up.

**Intervention:**

One-year, home-based vegetable gardening intervention providing gardening supplies and mentorship by cooperative extension–certified master gardeners to plant and maintain spring, summer, and fall gardens. Waitlisted participants received the identical intervention after 12 months.

**Main Outcomes and Measures:**

The main outcome was a composite index of improvements in self-reported vegetable and fruit consumption, physical activity, and physical function corroborated by plasma α-carotene levels, accelerometry, and physical performance assessments, respectively.

**Results:**

Of 381 enrolled participants (mean [SD] age, 69.8 [6.4] years; range, 50-95 years; 263 [69.0%] female), 194 were assigned to the gardening intervention and 187 were waitlisted (attrition rates, 7.2% and 7.0%, respectively). Intent-to-treat analyses did not detect a significant improvement in the composite index of vegetable and fruit intake, moderate-vigorous physical activity, and physical function (intervention arm vs waitlisted arm, 4.5% vs 3.1%; *P* = .53) or between-arm differences in vegetable and fruit intake (mean difference, 0.3 [95% CI, −0.1 to 0.7] servings per day; *P* = .10). The intervention arm experienced a significant improvement in vegetable and fruit intake (mean increase, 0.3 [95% CI, 0.0-0.6] servings per day; *P* = .04). Significant improvements also were observed in the intervention arm vs waitlisted arm in physical performance (mean difference for 2-minute step test, 6.0 [95% CI, 0.8-11.2] steps; *P* = .03; for 30-second chair stand, 0.8 [95% CI, 0.1-1.5] repetitions; *P* = .02), perceived health (8.4 [95% CI, 3.0-13.9] points on a 100-point scale [higher scores indicate better health]; *P* = .003), and gut microbiome alpha diversity (84.1 [95% CI, 20.5-147.6] more observed species; *P* = .01). The COVID-19 pandemic significantly moderated effects (eg, odds of improvement in self-reported physical functioning were greater before vs during the pandemic: odds ratio, 2.17; 95% CI, 1.12-4.22; *P* = .02).

**Conclusions and Relevance:**

In this randomized clinical trial including older cancer survivors, a vegetable gardening intervention did not significantly improve a composite index of diet, physical activity, and physical function; however, survivors assigned to the intervention had significantly increased vegetable and fruit consumption and, compared with waitlisted survivors, experienced significant improvements in perceived health and physical performance. Further study in broader populations and during pandemic-free periods is needed to determine definitive benefits.

**Trial Registration:**

ClinicalTrials.gov Identifier: NCT02985411

## Key Points

**Question:**

Does vegetable gardening improve health outcomes in older cancer survivors?

**Findings:**

In this randomized clinical trial including 381 cancer survivors aged 50 years or older across Alabama, a vegetable gardening intervention did not significantly improve a composite index of diet, physical activity, and physical function; survivors assigned to the intervention had significantly increased vegetable and fruit consumption and, compared with waitlisted survivors, experienced significant improvements in physical performance.

**Meaning:**

Vegetable gardening improved some health outcomes among cancer survivors, warranting further study in pandemic-free times.

## Teaser

This randomized clinical trial investigates whether vegetable gardening improves vegetable and fruit consumption, physical activity, and physical function outcomes among Medicare-eligible cancer survivors.

## Introduction

The benefits of gardening were first reported by Cicero in 46 BC.^1^ More recently, a meta-analysis of 1046 older adults from 13 randomized clinical trials (RCTs) and 2 cohort studies found that gardening was associated with improved physical functioning, increased physical activity, reduced body mass index (BMI), and improved quality of life.^2^ A systematic review also found that access to one’s own vegetable garden was an environmental factor associated with vegetable and fruit intake.^3^ Thus, gardening, especially vegetable gardening, may be associated with holistic health benefits and address the wide-ranging needs of cancer survivors at risk for a second malignant tumor, obesity, cardiovascular disease, other comorbidities, and functional impairment.^4,5,6^

Over a decade ago, the Harvest for Health feasibility trial was conducted among 12 survivors of breast, prostate, and childhood cancers residing in Jefferson County, Alabama.^7^ It was a partnered effort between an academic medical center (which recruited and enrolled participants and assessed changes in diet, physical activity, and physical functioning) and the Auburn University Horticulture Extension Office cooperative extension (which deployed extension-certified master gardeners [EMGs] to provide biweekly mentorship on planning, planting, and caring for a spring, summer, and fall raised-bed garden at participants’ homes). The trial demonstrated full accrual within a week, 83.3% retention over 1 year, and safety.^7^ Preintervention and postintervention assessments suggested improvements in physical performance,^8^ increased vegetable and fruit intake, and increased moderate-to-vigorous physical activity (MVPA).^7^ The study was expanded to 82 breast cancer survivors residing in metropolitan Birmingham, Alabama, and then statewide to 46 older survivors of various cancers using a 2-arm RCT design with comparison against a waitlist control.^9^ Both trials evaluated the Harvest for Health intervention, which was modified to supply either raised beds or 4 grow boxes of comparable square footage to increase reach to survivors who resided in condominiums or rental properties. Findings of both trials demonstrated feasibility (ie, 82%-100% accrual, 91%-95% retention, absence of serious attributable adverse events, and 100% “good-to-excellent” satisfaction). The trial among breast cancer survivors found significantly greater improvements in the intervention vs waitlisted arms in MVPA (114 vs −17 minutes per week), 2-minute step test (122 vs 110 steps), and arm curl test (12.7 vs 10.1 repetitions),^10^ whereas the RCT among older survivors found significantly attenuated increases in waist circumference (2.30 cm vs 7.96 cm).^11^ Both feasibility studies found trends toward higher vegetable and fruit intake.

These positive findings, plus those from survey studies,^12,13^ observational studies,^14^ and gardening interventions in other populations,^2,15,16,17,18,19,20,21^ reinforce that adequately powered trials to formally test the benefits of gardening interventions among cancer survivors are needed. Herein, we report the results of the fully powered Harvest for Health RCT that was tested among Medicare-eligible cancer survivors across Alabama.

## Methods

Harvest for Health was a 2-arm, single-blinded RCT that used a waitlist-controlled, crossover design to test the effects of a home-based gardening intervention on vegetable and fruit intake, physical activity, physical functioning, quality of life, sleep, adiposity, gut microbiome, and inflammatory biomarkers. Individuals assessing outcomes and conducting analyses were blinded to arm assignment. This study was approved by the University of Alabama at Birmingham (UAB) institutional review board (NCT02985411; the trial protocol is given in Supplement 1). All study participants provided written informed consent. We followed the Consolidated Standards of Reporting Trials (CONSORT) reporting guideline. The study period spanned May 11, 2016, to May 2, 2022, with detailed methods and recruitment results published previously.^22^

### Participants

Medicare-eligible cancer survivors who were diagnosed with and had completed curative therapy for cancers with 5-year survival of 60% or more^22^ were ascertained through UAB or Alabama state cancer registries and approached via direct mail^23^ followed by telephone contact. Referrals responding to media also were enrolled after verification of diagnoses by health care practitioners. Interested survivors were screened for suboptimal vegetable and fruit consumption (<5 servings per day), physical activity (<150 MVPA minutes per week), physical functioning (36-Item Short Form Health Survey [SF-36] subscale score ≤90 [score range, 0-100, with higher scores indicating better physical functioning]), and living independently at a residence that could accommodate 1 raised bed (1.2 × 2.4 m) or 4 grow boxes (62.2 × 52.1 cm) with 6 or more hours per day of sunshine and ready access to water. Other criteria were ability to speak, read, and write English and willingness to participate for 2 years with randomization to either study arm. Exclusion criteria included vegetable gardening within the past 2 years or medical conditions that precluded gardening. All study participants were followed up from initial contact until study completion or termination according to the CONSORT guideline.^24^

### Assessments

Assessments occurred at baseline (before randomization) and 12 months and included anthropometric measures (height [baseline only], weight, and waist circumference),^25^ physical performance testing (Senior Fitness Battery^8^), accelerometry (7-day collection via GTPX3 accelerometer [ActiGraph]), the Community Healthy Activities Model Program for Seniors (CHAMPS) Physical Activity Questionnaire for Older Adults,^26^ blood collection via venipuncture (after ≥4 hours of fasting), and collection of toenail clippings and a fecal wipe. Plasma and serum samples and fecal wipes were stored at −80 °C and toenails at room temperature^27^ and then batch-analyzed following published methods for α-carotene,^28^ cortisol,^29^ fecal microbiome,^30,31,32,33^ and inflammatory biomarkers.^22^ A remote protocol was implemented in April 2020 due to the COVID-19 pandemic. It omitted grip strength, arm curl, and gait speed measures from the performance battery because of cost or failure to prove reliability,^22,34^ and dried blood spots (found valid and reliable for interleukin-6 [IL-6]) and tumor necrosis factor–α [TNF-α]) supplanted venipuncture. Assessment of plasma α-carotene levels (an objective measure of vegetable and fruit intake) was not possible. Surveys administered online (REDCap) or by mail captured self-reported race, ethnicity, comorbidities, and symptoms^35^; falls^36^; perceived stress^37^; reassurance of worth^38^; sleep quality^39^; vegetable and fruit consumption^40^; physical activity^26^; quality of life^41^; and changes in health. Race and ethnicity were included in the analysis as potential effect modifiers; categories were African American or Black, non-Hispanic White, and other (included American Indian, Asian, Pacific Islander, Hispanic, and multiracial; combined into 1 category because <2% of participants identified as a race other than African American or Black or White and/or as Hispanic ethnicity).

### Randomization and Intervention

After conclusion of the baseline assessment, a staff member opened a sealed envelope (created by a statistician) to reveal the randomization assignment. Participants were evenly allocated within each county to receive the intervention immediately or to be waitlisted using computer-generated permuted block randomization. In the intervention group, the EMGs were paired with cancer survivors within counties of residence and visited survivors’ homes monthly to help establish and then maintain a spring, summer, and fall garden. Telephone, email, or text messages were scheduled between visits, with social cognitive theory undergirding all communications.^42^ The EMGs logged all encounters and provided time-stamped garden photographs. Each participant received a 1.2 × 2.4-m raised bed kit (or 4 grow boxes), soil, fertilizer, mulch, frost cloths, tomato cages, trellises, gardening hose, watering can, trowel, cultivator, soft chemistry insect control products, seeds, transplants, and a gardening journal. Participants and EMGs received hats, sunscreen, and binders with contact information, interaction tracking logs, and publications on gardening, safety, health, and vegetables and fruits (including recipes). Supplies were distributed at EMG-participant meet-and-greets (informational sessions and social events) at local community centers before initial planting. During the COVID-19 pandemic, meetings were conducted via videoconferencing and supplies were distributed in parking lots; all EMG-participant interactions were restricted to masked, outdoor encounters. Waitlisted participants received the identical intervention after 12 months.

### Statistical Analysis

A composite dichotomous score (yes or no) served as the primary outcome based on baseline-to-12-month attainment of the following benchmarks: increased consumption of 1 or more vegetable and fruit servings per day, corroborated by an increase of at least 10% in plasma α-carotene level^43^; 30 or more minutes of MVPA per week, corroborated by accelerometry; and increase of at least 5 SF-36 physical functioning subscale points, corroborated by improved scores in at least 60% of physical performance tests. Power calculations indicated that 185 participants per arm would yield more than 90% power to detect between-arm differences using χ^2^ testing (assumptions: 15% attrition and a moderate response difference ≥20%).^44^

Descriptive statistics and normality checks were performed on continuous variables (results from transformed analyses were similar). All statistical tests were 2-sided (α < .05) and conducted using SAS, version 9.4 (SAS Institute Inc); an intent-to-treat approach was used for all analyses.

Continuous outcomes were explored using linear mixed-effects models, including fixed effects for group, time, and group × time interaction, with a random intercept for participants estimated using restricted maximum likelihood. The mean change at 12 months vs baseline within each group and the difference in mean change between groups, with corresponding 95% CIs, were reported.

Categorical secondary outcomes (weight, waist circumference, health-related quality of life, reassurance of worth [Revised Social Provision Scale], self-efficacy to garden, social support to garden, sleep quality, perceived stress, cortisol level, IL-6 level, TNF-α level, and microbiome alpha diversity) were explored using logistic mixed-effects models with the same aforementioned model terms and assumed a binary or multinomial response distribution. Odds ratios (ORs) and their corresponding 95% CIs were reported.

Linear mixed-effects models are robust against missing data, as they can accommodate unbalanced data patterns^45,46^; thus, all available observations and participants were included in the analysis. For each outcome, participants with at least 1 nonmissing outcome measure were included in the analysis.

Logistic regression analyses were performed to determine whether garden type (raised beds or grow boxes) and EMG contact of 90% or more vs less than 90% modified the effects. Other potential effect modifiers (cancer type [breast and gynecologic cancer vs others], time elapsed from diagnosis [<5 years vs ≥5 years], age [<70 years vs ≥70 years], sex, race and ethnicity [non-Hispanic White as the reference group], educational attainment [high school graduate or less vs above], household size [1 vs ≥2 members], annual income [<$60 000 vs ≥$60 000], rural or urban county of residence, BMI [<30 vs ≥30; calculated as weight in kilograms divided by height in meters squared], vitality [<70% vs ≥70% of the SF-36 subscale score],^41^ garden start season [spring vs others], and trial completion before vs during the COVID-19 pandemic) were explored similarly.

## Results

This trial randomized 381 survivors of a variety of cancers with dates of diagnosis preexisting enrollment by 2 to 43 years and ages spanning 50 to 95 years (mean [SD] age, 69.8 [6.4] years) to Harvest for Health (194 participants) or the waitlist control (187 participants) (Table 1). A total of 263 participants (69.0%) were female, and 118 (31.0%) were male; 67 (17.6%) were African American or Black; 296 (77.7%), non-Hispanic White; 7 (1.8%), other race and ethnicity; and 11 (2.9%), unknown race and ethnicity. Most were married, urban dwelling, and currently retired or unemployed. A total of 218 (57.2%) were college graduates, and 94 (24.7%) reported an annual income less than $30 000. While few survivors were current smokers, 310 (81.4%) had a BMI of 25 or greater, and participants ate a mean (SD) of 2.1 (1.7) servings of fruits and vegetables a day, less than half of the recommended amount.^47^ While participants were screened with 1 item to verify suboptimal physical activity, results of accelerometry and the longer CHAMPS Physical Activity Questionnaire at the baseline assessment revealed higher physical activity levels.^26^

**Table 1.** Characteristics of the Harvest for Health Study Sample

Characteristic	Participants, No. (%)		
	All (N = 381)	Intervention (n = 194)	Control (n = 187)
Age, mean (SD), y	69.8 (6.4)	70.3 (6.6)	69.4 (6.2)
Cancer type^a^			
Breast	149 (39.1)	72 (37.1)	77 (41.2)
Genitourinary	75 (19.7)	37 (19.1)	38 (12.3)
Gynecologic	48 (12.6)	25 (12.9)	23 (20.3)
Alimentary	47 (12.3)	26 (13.4)	21 (11.2)
Other^b^	62 (16.3)	34 (17.5)	28 (15.0)
Cancer treatment			
Surgery	299 (78.5)	154 (79.4)	145 (77.5)
Radiation	150 (39.4)	84 (43.3)	66 (35.3)
Chemotherapy	6 (1.6)	0	6 (3.2)
Hormonal therapy	79 (20.7)	39 (20.1)	40 (21.4)
None of these or unknown	41 (10.8)	22 (11.3)	19 (10.2)
Time since diagnosis, mean (SD), mo	90.1 (53.9)	89.3 (49.6)	91.1 (58.3)
Sex			
Female	263 (69.0)	133 (68.6)	130 (69.5)
Male	118 (31.0)	61 (31.4)	57 (30.5)
Race and ethnicity			
African American or Black	67 (17.6)	36 (18.6)	31 (16.6)
Non-Hispanic White	296 (77.7)	148 (76.3)	148 (79.1)
Other^c^	7 (1.8)	3 (1.6)	4 (2.1)
Unknown or declined to answer	11 (2.9)	7 (3.6)	4 (2.1)
Educational level			
<High school	9 (2.4)	5 (2.6)	4 (2.1)
High school diploma	52 (13.7)	28 (14.4)	24 (12.8)
Some college or trade school	102 (26.8)	52 (26.8)	50 (26.7)
College graduate or postgraduate	218 (57.2)	109 (56.2)	109 (58.3)
Currently employed	101 (26.5)	47 (24.2)	54 (28.9)
Annual household income, $			
<30 000	94 (24.7)	47 (24.2)	47 (25.1)
30 000 to <60 000	102 (26.8)	54 (27.8)	48 (25.7)
≥60 000	141 (37.0)	70 (36.1)	71 (38.0)
Unknown or declined to answer	44 (11.5)	23 (11.9)	21 (11.2)
Household size, No. of members			
1	99 (26.3)	50 (26.2)	49 (26.3)
2	223 (59.2)	111 (58.2)	112 (60.2)
≥3	55 (14.6)	30 (15.7)	25 (13.4)
Marital status			
Married	233 (61.2)	119 (61.3)	114 (61.0)
Widowed	63 (16.5)	30 (15.5)	33 (17.6)
Other	85 (22.3)	45 (23.2)	40 (21.4)
Rural residence	44 (11.6)	24 (12.4)	20 (10.7)
Comorbidities, mean (SD) [range], No.	4.2 (2.5) [0-14]	4.4 (2.6) [0-13]	4.0 (2.4) [0-14]
Functional limitations reported, mean (SD) [range], No.^d^	4.8 (2.9) [0-10]	5.1 (2.9) [0-10]	4.4 (2.8) [0-10]
Current smoker	18 (4.7)	9 (4.6)	9 (4.8)
BMI^e^			
Normal	64 (17.1)	28 (14.7)	36 (19.6)
Overweight	123 (32.9)	64 (33.7)	59 (32.1)
Obese	187 (50.0)	98 (51.6)	89 (48.4)
Moderate-to-vigorous physical activity, mean (SD), min/wk	212 (355)	182 (276)	244 (421)
Vegetable and fruit intake, mean (SD), servings/d	2.1 (1.7)	2.0 (1.7)	2.1 (1.8)

Abbreviation: BMI, body mass index (calculated as weight in kilograms divided by height in meters squared).

^a^ Restricted to participants with 5-year survival rates of 60% or greater (in situ or localized cancers of the bladder or cervix, gastric cardia, or early-stage multiple myeloma; in situ or locoregionally staged melanoma and cancers of the colorectum, endometrium, kidney or renal pelvis, oral cavity or pharynx, ovary, prostate, thyroid, or female breast; and all stages of testes cancer, leukemia, and Hodgkin or non-Hodgkin lymphoma).

^b^ Lymphoma, thyroid, head and neck, melanoma, or multiple myeloma.

^c^ Included American Indian, Asian, Pacific Islander, Hispanic, or multiracial. These were combined into 1 category because less than 2% of participants identified as a race other than African American or Black or White and/or as Hispanic ethnicity.

^d^ According to the 36-Item Short Form Health Survey physical function subscale.

^e^ Normal was defined as 18.5 to less than 25.0, overweight as 25.0 to less than 30.0, and obese as greater than or equal to 30.0.

**Figure 1.**
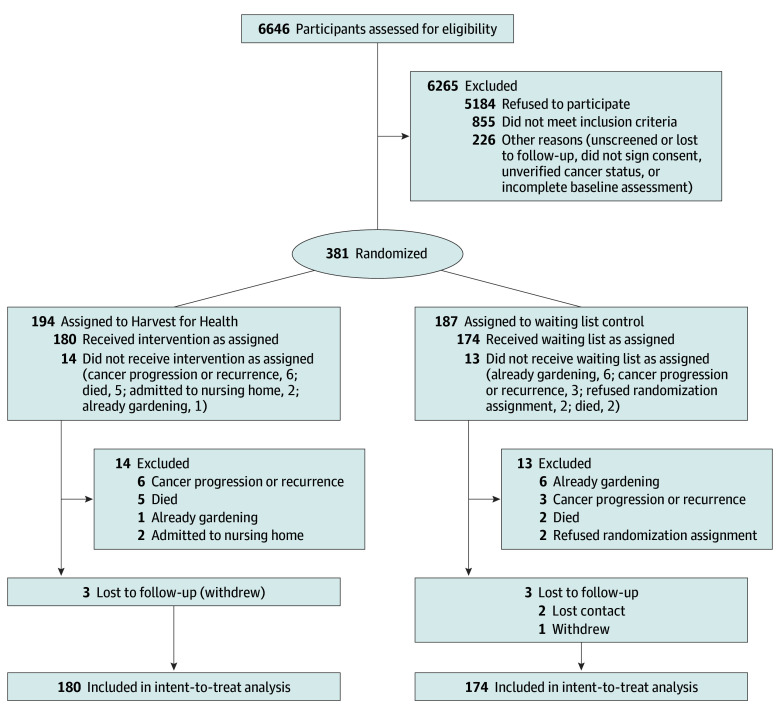
Harvest for Health CONSORT Diagram

Details on enrollment and postrandomization events are shown in Figure 1. Among the older cancer survivors participating in this trial during the COVID-19 pandemic, 14 (7.2%) in the intervention arm and 13 (7.0%) in the waitlisted arm were excluded before receiving their full assigned study condition. These postrandomization exclusions were evenly distributed between arms, with approximately one-quarter of these participants being excluded due to cancer progression (intervention, 6 [3.1%]; control, 3 [1.1%]), another quarter due to death (intervention, 5 [2.6%]; control, 2 [1.1%]), and another quarter due to subsequently divulging that they were gardening at baseline (intervention, 1 [0.5%]; control, 6 [3.2%]). Two participants (1.1%) assigned to the waitlist refused their randomization assignment. Three participants in each arm (intervention, 1.5%; control, 1.6%) withdrew or were lost to follow-up; thus, the intent-to-treat analysis included a sample of 180 in the intervention arm and 174 who were waitlisted. No between-arm differences in dropouts or adverse events were detected (the only attributable adverse event was nonserious minor bruising from a fall occurring while harvesting vegetables).

Approximately one-half of the intervention arm elected to receive raised beds (99 [51.0%]), whereas 95 (49.0%) preferred grow boxes; amounts of all other supplies were identical. Mean (SD) monthly adherence to EMG contact was 83.7% (20.2%).

**Figure 2.**
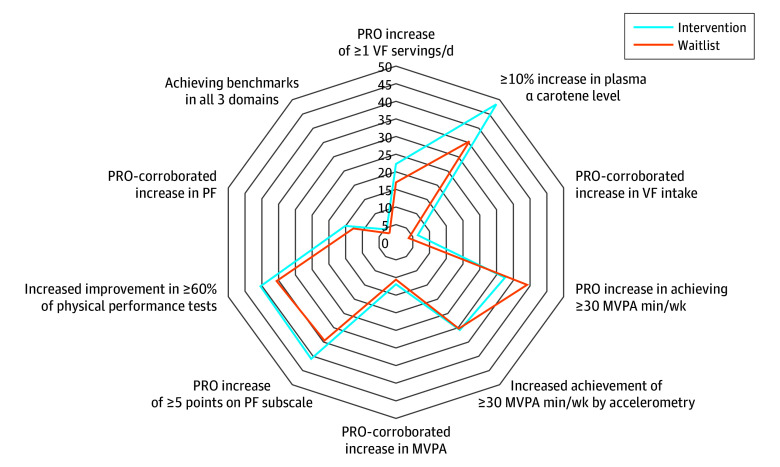
Percentages of Survivors in Each Arm Who Demonstrated Benchmark Improvements in Primary Outcomes Benchmark levels of objective and patient-reported outcomes (PROs) included increases in self-reported vegetable and fruit (VF) consumption of 1 or more servings per day, corroborated by a 10% increase or more in plasma α-carotene level; increases in self-reported moderate-to-vigorous physical activity (MVPA) of 30 or more minutes per week, corroborated by equivalent increases in accelerometry-assessed MVPA; and increases of 5 or more points on the 36-Item Short Form Health Survey physical function (PF) subscale, corroborated by improvements in at least 60% of physical performance tests.

Figure 2 depicts percentages of survivors in each arm who demonstrated benchmark improvements in primary outcomes; the intent-to-treat analysis did not detect a significant improvement in the composite index of vegetable and fruit intake, physical activity, and physical function (intervention arm vs waitlisted arm, 4.5% vs 3.1%; *P* = .53). With the exception of self-reported MVPA, greater baseline-to-12-month improvements were observed in the intervention arm compared with the waitlisted arm. These data showed consistent directionality, although no significant between-arm differences were detected. Table 2 provides continuous data on each of these variables. There were several significant within- and between-group differences. For example, a significant improvement in daily servings of vegetables and fruits was detected in the intervention arm (mean change, 0.3 servings; 95% CI, 0.0-0.6 servings; *P* = .04), whereas no difference was observed among controls (mean change, 0.0 servings; 95% CI, −0.3 to 0.2 servings; *P* = .76); plasma α-carotene levels paralleled these values. While no significant between-arm differences were detected in vegetable and fruit consumption (mean difference, 0.3 servings per day; 95% CI, −0.1 to 0.7 servings per day; *P* = .10), between-arm differences were found for both the 2-minute step test (significant decline in waitlisted arm: −5.4 steps [95% CI, −9.1 to −1.7 steps]; mean between-group difference, 6.0 steps [95% CI, 0.8-11.2 steps]; *P* = .03) and the 30-second chair-stand test (significant improvement in intervention arm: 0.6 repetitions [95% CI, 0.2-1.1 repetitions]; mean between-group difference, 0.8 repetitions [95% CI, 0.1-1.5 repetitions]; *P* = .02). These results were consistent regardless of EMG contact. However, there was evidence of significant effect moderation. In analyses of the intervention arm alone, participants who were more proximal to diagnosis demonstrated greater gains in physical functioning (odds ratio [OR], 0.37; 95% CI, 0.16-0.86; *P* = .02) and those reporting higher baseline vitality showed larger improvements in MVPA (OR, 2.21; 95% CI, 1.05-4.66; *P* = .04). The only variable that significantly moderated effects within the entire sample was the COVID-19 pandemic, for which significantly greater odds of improvements in self-reported physical functioning (difference of 5 points) were observed among participants who completed the study before vs during the pandemic (OR, 2.17; 95% CI, 1.12-4.22; *P* = .02).

**Table 2.** Primary Outcome Variables in the Harvest for Health Trial in Older Cancer Survivors

Outcome	Value, mean (SD)				Estimated least squares change (12 mo − baseline)^a^				Absolute difference in change, intervention vs control^a^	
	Intervention (n = 180)		Control (n = 174)		Intervention (n = 180)		Control (n = 174)			
	Baseline	12 mo	Baseline	12 mo	Mean (95% CI)	*P* value	Mean (95% CI)	*P* value	Mean (95% CI)	*P* value
VF intake, servings/d^b^	2.1 (1.7)	2.3 (1.8)	2.1 (1.8)	2.0 (1.6)	0.3 (0.0 to 0.6)	.04	0.0 (−0.3 to 0.2)	.76	0.3 (−0.1 to 0.7)	.10
Plasma α-carotene level, μmol/L^c^	0.1 (0.1)	0.1 (0.1)	0.1 (0.1)	0.1 (0.1)	0.0 (0.0 to 0.0)	.35	0.0 (0.0 to 0.0)	.79	0.0 (0.0 to 0.0)	.40
MVPA, min/wk^d^	187.8 (281.5)	218.0 (382.2)	255.0 (432.8)	235.4 (375.5)	31.7 (−28.9 to 92.0)	.30	−14.2 (−74.2 to 45.9)	.64	45.8 (−39.3 to 131.0)	.29
Accelerometry MVPA, min/wk	214.9 (194.1)	207.0 (200.7)	209.4 (150.3)	239.3 (300.9)	−8.2 (−45.0 to 28.6)	.66	31.8 (−4.7 to 68.2)	.09	−40.0 (−91.8 to 11.8)	.13
SF-36 physical function score^e^	66.1 (22.7)	67.0 (25.0)	70.1 (22.5)	68.5 (24.1)	1.2 (−1.6 to 3.9)	.42	−2.1 (−4.9 to 0.7)	.15	3.2 (−0.7 to 7.2)	.11
Physical performance										
2-min Step test, steps, No.	76.9 (24.7)	77.5 (25.3)	81.5 (25.8)	75.9 (27.0)	0.6 (−3.1 to 4.2)	.75	−5.4 (−9.1 to −1.7)	.005	6.0 (0.8 to 11.2)	.03
8-ft Timed Up and Go test, s	8.6 (2.9)	8.4 (3.1)	8.5 (4.1)	8.5 (3.2)	−0.1 (−0.5 to 0.3)	.58	0.3 (−0.1 to 0.7)	.17	−0.4 (−1.0 to 0.2)	.17
Chair stand, repetitions in 30 s, No.	9.8 (3.8)	10.4 (4.0)	10.1 (4.2)	10.0 (4.3)	0.6 (0.2 to 1.1)	.008	−0.1 (−0.6 to 0.3)	.55	0.8 (0.1 to 1.5)	.02
Chair sit and reach, cm	−2.9 (10.5)	−3.8 (10.6)	−2.0 (10.5)	−2.5 (11.0)	−0.8 (−2.3 to 0.8)	.32	−0.3 (−1.9 to 1.3)	.70	−0.5 (−2.7 to 1.8)	.67
Back scratch, cm	−12.6 (12.5)	−13.6 (12.8)	−13.0 (13.7)	−12.5 (13.5)	−0.7 (−2.6 to 1.1)	.43	0.4 (−1.5 to 2.3)	.66	−1.2 (−3.8 to 1.5)	.39

Abbreviations: MVPA, moderate-to-vigorous physical activity; SF-36, 36-Item Short Form Health Survey; VF, vegetable and fruit.

^a^ Established from linear mixed-effects models.

^b^ Based on the Eating at America’s Table Study.

^c^ Only assessed among participants who completed the trial prior to the onset of the COVID-19 pandemic (95 in the intervention arm and 80 in the waitlisted arm).

^d^ Based on the Community Healthy Activities Model Program for Seniors Physical Activity Questionnaire for Older Adults.

^e^ Scores range from 0 to 100, with higher scores indicating better functioning.

Table 3 provides secondary outcome data. A modest weight loss of 0.8 kg over 12 months was observed with the gardening intervention but not among controls (mean between-arm difference, −1.0 kg; 95% CI, −2.2 to 0.1 kg; *P* = .08). Significant between-arm differences were noted for perceived health, which improved in the intervention arm but not among controls (8.4 points [95% CI, 3.0-13.9 points] on a 100-point scale, with higher scores indicating better health; *P* = .003). Other quality-of-life outcomes (eg, physical and emotional role and general health) improved in both arms, with no between-arm differences. Inflammatory markers (cortisol, IL-6, and TNF-α) remained fairly stable over the study period in both groups. Likewise, no significant between-arm differences were found for gardening self-efficacy (a hypothesized intervention mediator); however, significant improvements in social support to garden (another hypothesized mediator) were detected in the intervention arm (mean [SD] days of support per month, 4.5 [6.0] at baseline vs 9.0 [7.7] at 12 months; mean change, 4.4 days [95% CI, 3.3-5.6 days]; *P* < .001). Between-arm differences also were detected for microbiome alpha diversity (observed species: 84.1 [95% CI, 20.5-147.6] more in the intervention group; *P* = .01), with significant declines observed among waitlisted survivors across 3 other indices (whole tree phylogeny, Shannon, and Simpson indices).

**Table 3.** Secondary Outcomes in the Harvest for Health Trial in Older Cancer Survivors

Outcome	Value, mean (SD)				Estimated least squares change (12 mo − baseline)^a^				Absolute difference in change for intervention vs control^a^	
	Intervention (n = 180)		Control (n = 174)		Intervention		Control			
	Baseline	12 mo	Baseline	12 mo	Mean (95% CI)	*P* value	Mean (95% CI)	*P* value	Mean (95% CI)	*P* value
Weight, kg	85.6 (17.8)	84.7 (17.0)	85.0 (19.0)	84.8 (18.8)	−0.8 (−1.6 to 0.0)	.04	0.2 (−0.6 to 1.0)	.66	−1.0 (−2.2 to 0.1)	.08
Waist circumference, cm	102.4 (14.4)	101.7 (14.1)	101.5 (13.5)	102.1 (15.0)	−0.6 (−1.7 to 0.5)	.32	0.9 (−0.2 to 2.0)	.11	−1.4 (−3.0 to 0.1)	.07
Health-related QOL score										
Physical health composite^b^	59.1 (17.1)	65.9 (20.0)	62.1 (17.1)	67.3 (18.6)	7.0 (4.8 to 9.1)	<.001	4.9 (2.8 to 7.1)	<.001	2.0 (−1.0 to 5.1)	.19
Physical role	49.9 (21.2)	67.7 (26.3)	53.2 (20.4)	68.2 (25.9)	17.9 (14.6 to 21.3)	<.001	14.9 (11.5 to 18.3)	<.001	3.0 (−1.7 to 7.8)	.21
Pain	65.9 (22.4)	66.1 (23.2)	68.1 (22.3)	68.2 (22.9)	0.6 (−2.4 to 3.5)	.70	−0.3 (−3.3 to 2.7)	.86	0.8 (−3.4 to 5.1)	.69
General health	54.8 (18.5)	62.7 (19.6)	57.0 (19.4)	64.2 (19.3)	8.1 (5.4 to 10.8)	<.001	7.4 (4.7 to 10.2)	<.001	0.6 (−3.2 to 4.5)	.74
Emotional health composite	68.2 (17.0)	73.1 (19.7)	70.8 (15.6)	75.2 (17.0)	4.9 (2.7 to 7.1)	<.001	4.2 (2.0 to 6.4)	<.001	0.7 (−2.4 to 3.8)	.66
Emotional well-being	78.3 (16.6)	77.6 (18.4)	79.9 (15.1)	78.8 (16.3)	−0.6 (−2.6 to 1.5)	.58	−1.1 (−3.1 to 1.0)	.30	0.5 (−2.3 to 3.4)	.72
Emotional role	61.1 (17.7)	79.3 (24.7)	63.4 (16.7)	80.5 (24.3)	18.1 (14.9 to 21.4)	<.001	17.1 (13.4 to 20.4)	<.001	1.0 (−3.6 to 5.7)	.66
Vitality	54.7 (20.9)	56.0 (21.3)	58.5 (18.8)	57.6 (19.5)	1.4 (−1.1 to 3.9)	.49	−0.9 (−3.5 to 1.6)	.46	2.3 (−1.2 to 5.9)	.20
Social functioning	78.5 (24.8)	79.4 (25.5)	81.6 (24.3)	83.7 (22.2)	0.7 (−3.0 to 4.5)	.70	1.9 (−1.9 to 5.8)	.33	−1.2 (−6.6 to 4.2)	.67
Perceived health^c^	55.3 (22.1)	60.2 (18.9)	58.2 (23.3)	54.5 (21.3)	4.9 (1.1 to 8.8)	.01	−3.5 (−7.4 to 0.4)	.08	8.4 (3.0 to 13.9)	.003
Reassurance of worth, SPS	13.4 (2.1)	13.3 (1.9)	13.6 (2.1)	13.6 (2.1)	0.0 (−0.4 to 0.3)	.77	0.2 (−0.3 to 0.3)	.13	−0.1 (−0.5 to 0.4)	.76
Sure to very sure of self-efficacy to maintain a thriving garden, No./total No. (%)	140/180 (77.8)	128/176 (72.7)	142/174 (81.6)	138/171 (80.7)	0.8 (0.5 to 1.2)^d^	.27	0.9 (0.5 to 1.6)^d^	.82	0.7 (0.5 to 1.1) ^d^	.09
Social support to garden, d/mo	4.5 (6.0)	9.0 (7.7)	4.8 (6.1)	4.5 (6.3)	4.4 (3.3 to 5.6)	<.001	−0.2 (−1.3 to 0.9)	.74	4.6 (3.0 to 6.2)	<.001
PSQI sleep quality score	7.3 (4.0)	6.9 (4.0)	7.1 (3.5)	6.8 (3.9)	−0.4 (−0.8 to 0.0)	.08	−0.3 (−0.7 to 0.1)	.18	−0.1 (−0.7 to 0.5)	.78
Perceived stress^e^	11.3 (7.2)	11.0 (7.0)	10.5 (6.9)	10.5 (6.9)	−0.4 (−1.3 to 0.4)	.31	0.1 (−0.8 to 0.9)	.43	−0.5 (−1.7 to 0.7)	.38
Toenail cortisol level, nmol/g	0.1 (0.3)	0.1 (0.2)	0.1 (0.2)	0.1 (0.2)	0.0 (−0.1 to 0.1)	.78	0.0 (−0.1 to 0.1)	.82	0.0 (−0.1 to 0.1)	.74
IL-6 level, pg/mL	1.8 (1.8)	1.7 (1.4)	1.5 (1.2)	1.5 (1.4)	−0.1 (−0.4 to 0.1)	.29	0.0 (−0.3 to 0.3)	.97	−0.1 (−0.5 to 0.3)	.49
TNF-α level, pg/mL	3.0 (1.6)	2.8 (1.2)	2.6 (1.5)	2.4 (0.9)	−0.2 (−0.4 to 0.1)	.22	−0.2 (−0.5 to 0.1)	.18	0.0 (−0.4 to 0.4)	.89
Microbiome alpha diversity										
Observed species, No.	198.9 (122.5)	222.4 (123.1)	245.9 (122.5)	187.1 (94.3)	25.1 (−25.7 to 76.0)	.33	−58.9 (−97.0 to −20.8)	.003	84.1 (20.5 to 147.6)	.01
Whole tree phylogeny index	19.7 (9.3)	22.2 (9.4)	23.5 (8.8)	19.5 (8.1)	2.6 (−1.3 to 6.5)	.19	−4.0 (−7.0 to −1.1)	.007	6.6 (1.7 to 11.5)	.008
Shannon index	5.1 (1.5)	5.3 (1.5)	5.6 (1.2)	4.8 (1.7)	0.2 (−0.5 to 0.9)	.52	−0.8 (−1.3 to −0.3)	.003	1.0 (0.2 to 1.9)	.02
Simpson index	0.9 (0.1)	0.9 (0.1)	0.9 (0.1)	0.8 (0.2)	0.0 (−0.1 to 0.1)	.82	−0.1 (−0.1 to 0.0)	.004	0.1 (0.0 to 0.2)	.06

Abbreviations: IL-6, interleukin 6; PSQI, Pittsburgh Sleep Quality Index; QOL, quality of life; SPS, Revised Social Provision Scale; TNF-α, tumor necrosis factor α.

^a^ Established from linear mixed-effects models.

^b^ The 36-Item Short Form Health Survey physical function subscale was a primary outcome; data are shown in Table 2.

^c^ On a 100-point scale, with higher scores indicating better health.

^d^ Odds ratio (95% CI).

^e^ Based on the Perceived Stress Scale.^37^

## Discussion

To our knowledge, this study is the largest RCT of a vegetable gardening intervention to date. While the Harvest for Health intervention did not have a significant effect on the composite primary outcome, it resulted in significant differences in individual components of the composite score and other secondary outcomes. For example, while the between-arm difference of 0.3 vegetable and fruit servings consumed per day in the current study falls below the difference of 0.5 to 1.2 servings per day reported in prior gardening interventions, including Harvest for Health pilot studies,^10,21,48^ it falls within the range of 0.1 to 1.4 servings per day reported in a systematic review of 44 intervention studies among adults that were focused exclusively on increasing vegetable and fruit intake.^49^ This variation could be due to the instruments used (vegetable and fruit screener vs food frequency questionnaires or dietary recalls) but more likely is attributable to shorter study periods and measures taken prior to planting vs harvest time as well as influences of seasonality, which have proven effects on vegetable and fruit consumption.^50^ While large meta-analyses indicated that overall mortality decreased with vegetable and fruit intakes of 5 servings per day,^51,52^ dose-response analyses suggested that larger health benefits may occur for increases from low to moderate intakes,^52,53^ as in the current study, in which baseline intakes averaged only 2.1 servings per day. Another community gardening intervention documented increases of 0.46 servings per day but did so in populations with baseline intakes of 4.95 servings per day^48^; in that study, Litt and colleagues^48^ excluded data collected after the COVID-19 pandemic on the premise that they may have differed from prepandemic values. Instead, the current study included all data, but as in the study by Litt et al,^48^ the lack of data on a primary outcome (α-carotene) was limiting, and results yielded hard evidence that another primary outcome (ie, physical functioning) was moderated by the COVID-19 pandemic.

Participants who completed the intervention prior to the pandemic had twice the likelihood of demonstrating a clinically meaningful physical functioning difference of 5 points.^54^ Changes during the study in factors associated with functional decline in older adults, such as pain, fatigue, or depression,^55,56,57,58^ whether or not related to the pandemic, could have affected an individual’s physical functioning, as reported in the Southwest Harvest for Health intervention.^18^ Also, physical activity may have contributed to COVID-19 pandemic–related moderation of physical functioning,^58^ even though the data suggested that physical activity was not moderated directly and there were no between-arm differences (given high heterogeneity). The absence of a statistically significant effect on MVPA mirrors findings of other studies^17,22,48^ and may relate to the need to assess populations with greater baseline vitality to observe postulated effects that the light-to-moderate activities of gardening serve as a gateway to more intensive exercise (eg, joining a gym); this also may have been affected by the pandemic.

Significant differences in the 2-minute step and 30-second chair-stand performance tests have been observed in other gardening studies, with increases in scores ranging from 4 to 35 steps and 0.6 to 1.4 stands, respectively.^10,21,22^ Our results for these 2 tests represent small, meaningful changes according to the effect size method.^44,59,60^ Importantly, these outcomes serve as significant factors associated with cardiorespiratory fitness,^61^ mobility,^62^ and frailty.^63^ Some physical performance tests associated with the Senior Fitness Battery that were used in prepandemic assessments, such as grip strength and arm curls (measures of upper-arm strength that improved in our group’s pilot studies^7,10^), were not possible during the COVID-19 pandemic and likely impaired the overall composite score.

Despite the pandemic and changes in protocol, perceived social support increased significantly across the study period among intervention arm participants. Therefore, despite restrictions placed on EMG-participant contact, the number of days per month that intervention arm participants reported receiving support doubled compared with unchanged levels among controls. Data were not as strong for self-efficacy, another key construct of social cognitive theory differences that might be further explored in future research.^64^

Finally, the intervention was found to have significant effects on key subjective (perceived health) and objective (gut microbiome alpha diversity) health-related outcomes by mitigating declines that typically occur with aging and chronic disease.^65,66^ Given associations of perceived health with mortality^66,67^ and of sustained higher levels of alpha diversity with decreasing risk of heart disease,^68^ diabetes,^69^ sarcopenia,^70^ and obesity^71^ (conditions that are prevalent among cancer survivors^4,5,6,65^), these effects are noteworthy.

### Strengths and Limitations

This study has strengths. It enrolled a diverse study sample in terms of cancer type, household income, and geography (rural and urban). Moreover, participants were fairly representative of older individuals in Alabama with respect to race and ethnicity (proportion of non-Hispanic White individuals in the study sample, 77.7%; Alabama, 73.9%) and income (Alabama’s median income among older adults is $47 114), though the proportions of female participants and college graduates in the study were higher (ie, 69.0% female [Alabama, 55%] and 57.2% college graduates [Alabama, 26.1%]).^72^ The trial also successfully enrolled and retained older cancer survivors up to 95 years of age with multiple comorbidities and functional limitations. This theoretically grounded study also included rigorous measures of health behaviors and outcomes that are important to cancer survivors, clinicians, and researchers alike.

The results should be interpreted in light of potential study limitations, which include bias resulting from a study sample with a higher educational level that was predominantly female, thus limiting generalizability. Moreover, the unblinded nature of the intervention may have influenced various behaviors or reporting. The COVID-19 pandemic also necessitated changes in intervention delivery and assessment and affected the results.

## Conclusions

In this RCT of a home-based vegetable gardening intervention among older cancer survivors, a vegetable gardening intervention did not significantly improve a composite index of diet, physical activity, and physical function; however, survivors assigned to the intervention had significantly increased vegetable and fruit consumption and, compared with waitlisted survivors, experienced significant improvements in perceived health, physical performance, and gut microbiome diversity. However, trial outcomes were significantly moderated by the COVID-19 pandemic. Thus, future studies are needed, especially those that use rigorous measures to evaluate the physical, mental, and social benefits of vegetable gardening and that explore potential mechanisms. Continued use and testing of an extant infrastructure for delivering the intervention (eg, a cooperative extension master gardener program) is warranted given the potential for widespread dissemination and sustainability.

## Notes

Supplement 1.
Trial Protocol

Supplement 2.
Data Sharing Statement
