# Supplementary material for: Vegetable Gardening and Health Outcomes in Older Cancer Survivors: A Randomized Clinical Trial
Source: JAMA Netw Open. 2024 Jun 20;7(6):e2417122. doi: 10.1001/jamanetworkopen.2024.17122 (PMC11190797; doi:10.1001/jamanetworkopen.2024.17122)
Supplement: Supplement 1. — Trial Protocol [file jamanetwopen-e2417122-s001.pdf]

Human Subjects Protocol (HSP)

Form Version: June 25, 2015

- **You are applying** for IRB review of the research described in this form.
- **To avoid delay**, respond to all items in order and include all required approvals and documents.
- **To complete the form**, click the underlined areas and type or paste in your text; double-click checkboxes to check/uncheck. For more tips, see www.uab.edu/irb/forms .
- **Mail or deliver all materials to AB 470**, 701 20th Street South, Birmingham, AL 35294-0104.

Indicate the type of review you are applying for:

☒ Convened (Full) IRB *or*

☐ Expedited—See the Expedited Category Review Sheet, and indicate the category(ies) here: ☐1 ☐2 ☐3 ☐4 ☐5 ☐6 ☐7

1. IRB Protocol Title: Harvest for Health in Cancer Survivors

2. Investigator, Contacts, Supervisors

a. Name of Principal Investigator: **Wendy Demark-Wahnefried** Degree(s)/Title: **PhD, RD**
BlazerID: **demark** Dept/Div: **Department of Nutrition Sciences**
Mailing Address: **WEBB 623. 1720 2nd Ave South, Birmingham, AL** UAB ZIP: **35294**
Phone: **205.975.4022** Fax: **205.975.2592** E-mail: **demark@uab.edu**

b. Name of Contact Person: **Dr. Wendy Demark-Wahnefried** Title: **Professor and Webb Endowed Chair of Nutrition Sciences; Associate Director of UAB Comprehensive Cancer Center** Phone: **205.975.4022** E-mail: **demark@uab.edu** Fax: **205.975.2592**
Mailing Address (if different from that of PI, above):______

INVESTIGATOR ASSURANCE STATEMENT & SIGNATURE

By my signature as Principal Investigator, I acknowledge my responsibilities for this Human Subjects Protocol, including:

- Certifying that I and any Co-Investigators or Other Investigators comply with reporting requirements of the UAB Conflict of Interest Review Board;
- Certifying that the information, data, and/or specimens collected for the research will be used, disclosed and maintained in accordance with this protocol and UAB policies;
- Following this protocol without modification unless (a) the IRB has approved changes prior to implementation or (b) it is necessary to eliminate an apparent, immediate hazard to a participant(s);
- Verifying that all key personnel listed in the protocol and persons obtaining informed consent have completed initial IRB training and will complete continuing IRB training as required;
- Verifying that all personnel are licensed/credentialed for the procedures they will be performing, if applicable;
- Certifying that I and all key personnel have read the *UAB Policy/Procedure to Ensure Prompt Reporting of Unanticipated Problems Involving Risks to Subjects or Others to the IRB, Institutional Officials*, *and Regulatory Agencies* and understand the procedures for reporting;
- Applying for continuing review of the protocol at least annually unless directed by the IRB to apply more frequently;
- Conducting the protocol as represented here and in compliance with IRB determinations and all applicable local, state, and federal law and regulations; providing the IRB with all information necessary to review the protocol; refraining from protocol activities until receipt of initial and continuing formal IRB approval.

Signature of Investigator: Wendy Demark-Wahnefried **Date: 5/11/2016**

3. Protocol Personnel

Including the PI, list all key personnel (each individual involved in the design and conduct of this protocol including recruitment, informed consent, analysis of the data, and reporting of the results). Complete either the UAB (3.a.) or non-UAB (3.b) table, as applicable. Use the checkboxes to show each person's role, whether the investigator has financial interests as defined by the UAB CIRB, and briefly describe the individual's responsibilities for the research and qualifications to perform those responsibilities. Insert additional rows as needed.
FDA: For studies involving investigational drugs, list all investigators who will be listed on FDA Form 1572 and include a copy of the 1572. Send the IRB a copy of Form 1572 any time you update the form with the FDA.

a. UAB Personnel

Name	Blazer ID	Role	Financial Interest?*	Protocol Responsibilities and Qualifications
Wendy Demark-Wahnefried	demark	Principal Investigator	☒ No<br>☐ Yes	Over 20 years of experience working with cancer survivors
Jennifer De Los Santos	drjfd	☒Sub-Investigator<br>☐Other	☒ No<br>☐ Yes	Radiation oncologist, pivotal for program recruitment
Meredith Kilgore	mkilgore	☒ Sub-Investigator<br>☐ Other	☒ No<br>☐ Yes	Expert in cost effectiveness investigation
Julie Locher	jlocher	☒ Sub-Investigator<br>☐ Other	☒ No<br>☐ Yes	Expert in community-based interventions aimed at older cancer survivors
Robert Oster	oster	☒ Sub-Investigator<br>☐ Other	☒ No<br>☐ Yes	Expert in study design and analysis
Yuko Tsuruta	tsuru	☐ Sub-Investigator<br>☒ Other	☒ No<br>☐ Yes	Staff in project intervention
Andrew Fruge	fruge	☐ Sub-Investigator<br>☒ Other	☒ No<br>☐ Yes	Staff in project intervention
Mallory Cases	mgcases	☐ Sub-Investigator<br>☒ Other	☒ No<br>☐ Yes	Staff in project intervention
Lora Roberson	lrob314	☐ Sub-Investigator<br>☒ Other	☒ No<br>☐ Yes	Staff in project intervention
Isabella Mak	imak	☐ Sub-Investigator<br>☒ Other	☒ No<br>☐ Yes	Staff in project intervention

*** Financial Interest** – for each investigator listed above, answer **Yes** or **No** as to whether the investigator or an immediate family member has any of the following:

- An ownership interest, stock options, or other equity interest related to the investigator's institutional responsibilities of any value.
- Compensation greater than $5,000 in the previous two years when aggregated for the immediate family
- Proprietary interest including, but not limited to, a patent, trademark, copyright, or licensing agreement.
- Board of executive relationship, regardless of compensation.
- Any other Financial Interest as defined by the UAB CIRB.

If the investigator has a Financial Interest, a disclosure has to be made to the UAB CIRB. A completed CIRB evaluation has to be available before the IRB will complete its review.

b. Non-UAB Personnel - Include individuals who will interact or intervene with participants, obtain consent, or have access to private, identifiable information for research purposes.

Name	Title	Do the Non-UAB personnel have their own IRB approval?	Financial Interest?*	Protocol Responsibilities and Qualifications
Name: Kerry Smith<br>Institution: Auburn University	☒ Sub-Investigator<br>☐ Other	☐ No - UAB IRB will determine if they are engaged in research.<br>☒ Yes - attach IRB approval	☒ No<br>☐ Yes	Team Co-Leader in the Alabama Cooperative Extension System
Name: Renee Thompson<br>Institution: Auburn University	☐ Sub-Investigator<br>☒ Other	☐ No - UAB IRB will determine if they are engaged in research.<br>☒ Yes - attach IRB approval	☒ No<br>☐ Yes	Team staff in the Alabama Cooperative Extension System

c. Do the investigators listed above include any students using this research for their thesis or dissertation?

☒ No, continue with Item 3.d.
☐ Yes, complete the following

Student Name	Thesis/Dissertation Title

d. Is the principal investigator a student, fellow, or resident? ☐Yes ☒No

If Yes, complete items below and obtain signature of faculty advisor or supervisor:

Supervisor's Name: ______
Degree(s) / Job Title: ______
Additional Qualifications pertinent to the study: ______
Telephone: ______
E-Mail: ______
Signature: ______________________________

e. Describe the principal investigator's activities related to this protocol and provisions made by the PI to devote sufficient time to conduct the protocol:

During the initial months of the developmental year, Dr. Demark-Wahnefried will work closely with various members of the research team, as well as subcontractors (Auburn investigators: Kerry Smith, MS and Renee Thompson) to fine tune data collection instruments and modify procedures for ordering and delivering garden supplies for the intervention. During subsequent months, Dr. Demark-Wahnefried will oversee data collection and management, and delivery of the intervention. She will conference routinely with the Auburn Cooperative Extension staff (as above), who will oversee the recruiting, training and support of the Master Gardener volunteers who agree to become "mentors" for the survivors in this intervention. Dr. Demark – Wahnefried will also conference routinely with the project manager. Dr. Demark-Wahnefried will be ultimately responsible for generating all progress reports, as well as the final report.

f. Is medical supervision required for this research? ☐Yes ☒No

If Yes, who will provide the supervision?

☐ PI will provide *-OR-* Name:______ Telephone:______

If other than PI, obtain signature of person providing medical supervision:

Signature ______________________________

g. Describe the process that ensures that all persons assisting with the research are adequately informed about the protocol and their research-related duties and functions:

All study staff who will be brought into the project over the five year-long study period will have received the required IRB training, as well as project specific training. Weekly meetings will be held to ensure that protocols are followed and that issues that arise are addressed in a timely fashion.

4. Funding

Is this study funded? ☒Pending ☐Yes ☐No

If No, specify that costs of the study will be covered by funds from the UAB department or other source named:

If Yes, attach one copy of completed application or request for funding sent to sponsor, and complete a-d.

a. Title of Grant or Contract: **Harvest for Health in Older Cancer Survivors 1 R01 CA201362-01A1**

b. PI of Grant or Contract: **Wendy Demark-Wahnefried**

c. Office of Sponsored Programs Proposal Number: **000511548**
(*or enter "Pending" and provide upon receipt from OSP*)

d. Sponsor, Funding Route (*check and describe all that apply*):

☒ Gov't Agency or Agencies—Agency name(s): **National Cancer Institute**
- ☐ Department of Defense (DoD): Identify DoD component:______
- ☐ Department of Energy (DOE)
- ☐ Department of Justice (DOJ)
- ☐ Department of Education

☐ NIH Coop. Group Trial—Group name:______

☐ Private Nonprofit (e.g., Foundation)—Name:______

☐ Industry, investigator-initiated—Name:______ Describe the funding arrangement:______
Note. Western IRB reviews industry-sponsored protocols unless the investigator initiated the research, or the study qualifies for expedited review or involves gene therapy.

☐ UAB Departmental/Division Funds—Specify:______

5. Locations Involved

a. Describe the facilities available for the conduct of the research. For research on UAB campus, include building names and room numbers:

Human subject contact will be performed via home visits. Roughly 10-12 days prior to the baseline visit, participants will be mailed questionnaires, a programmed accelerometer with instructions for a 7-day collection and a moistened wipe and collection bags for the stool and nail samples. These materials will be collected by study staff during the baseline visit conducted in participants' homes. Anthropometric measures, physical performance testing, blood draws, saliva collection and the physical activity recall at baseline and at follow up will be collected in participant's homes. Blood, stool, nail clipping and saliva samples will be stored in -80 degree freezers under back up emergency power at Volker Hall G005 and Wallace Tumor Institute 399S. Files will be stored and administrative duties will be performed in the Wallace Tumor Institute Room 102.

b. Indicate all "performance sites" that will provide space, services, facilities, potential or actual participants, or other support for this protocol.

☐ The Kirklin Clinic (TKC)
☐ University of Alabama Hospital (UAHosp)
☐ The Children's Hospital of Alabama (TCHA)
☐ Callahan Eye Foundation Hospital (CEFH)
☐ UAB Highlands

☐ Jefferson County Dept. of Health (JCDH)
☐ Birmingham Veterans Affairs Medical Center (BVAMC)
☐ General Clinical Research Center (GCRC)—inpatient
☐ General Clinical Research Center (GCRC)—outpatient
☐ General Clinical Research Center (GCRC) at The Kirklin Clinic (TKC)
☒ Other (i.e., Any performance site not listed above, including those covered by subcontracts related to this protocol)—Describe: **Participants' homes and samples stored at Volker Hall G005 and Wallace Tumor Institute 399S**

c. Is this study a clinical trial requiring clinical services at one of the performance sites listed in Item b above? ☐Yes ☒No
If Yes, Fiscal Approval Process (FAP)-designated units complete a FAP submission and send to fap@uab.edu. For more on the UAB FAP, see www.uab.edu/osp/clinical-billing-review.

d. Is this a field study? ☒Yes ☐No
If Yes, describe the community and include information about how the community will be involved in the design, implementation and analysis of the research. This would include focus groups, training local facilitators/community health advisors:

The study is a gardening intervention among 426 older cancer survivors in the state of Alabama. Survivors will be randomized to either a delayed intervention control group or immediate intervention group where they are paired with Cooperative Extension certified master gardeners to plant a vegetable garden at their place of residence (the intervention). Baseline, 1- and 2- years follow up testing will occur in the participant's home. Previous pilot work provides an established relationship with the Cooperative Extension as well as training mechanisms for the Master Gardeners.

e. Is the study to be undertaken within a school, business, or other institution that does not have an institutional review board? ☐Yes ☒No
If Yes, attach a statement of any contacts with and approvals from the appropriate institution officials.
Note. Documentation of all such approvals must be received by the UAB OIRB before IRB approval will be issued.

f. Has this protocol or project been reviewed by another IRB, similar review board, or departmental review committee(s) that authorizes the use of its patient populations? ☐Yes ☒No
If Yes, provide name of the review board(s):______ and for each board listed, enter either the date of latest approval(s) or "PENDING":______ or reasons not approved:______. *If this protocol is subsequently rejected or disapproved by another review board, the UAB IRB must be notified promptly.*
Attach copies of approvals/disapprovals.

g. Will any of the participants be from the Birmingham Veterans Affairs Medical Center? ☐Yes ☒No

If Yes, attach VA IRB approval or notification from the VA Research and Development Department that the study has been submitted to the VA IRB for review.

h. Will the study be conducted at or recruit participants from the Jefferson County Department of Public Health (JCDH)? ☐Yes ☒No
If Yes, attach notification that the protocol has been approved by JCDH or the Alabama Department of Public Health IRB.

6. Multi-Site Studies

a. Is the investigator the lead investigator of a multi-site study? ☐ Yes ☒No
b. Is UAB a coordinating site in a multi-site study? ☐Yes ☒No
c. If you answered **Yes** to *a* or *b*, describe the management of information obtained in multi-site research that might be relevant to the protection of participants. Include, at a minimum, the following items:

- o IRB approvals from other sites
- o Unanticipated problems involving risks to participants or others. (For example, if there is an unanticipated problem involving risks to participants or others, which site is responsible for reporting it?)
- o Interim results.
- o Protocol modifications.

______

7. Drugs: Will any drugs or supplements be used/studied in this protocol? ☐Yes ☒No
If Yes, attach the Drug Review Sheet.

8. Devices: Will any devices be studied in this protocol or used for a purpose other than for which they were approved by the FDA? ☐Yes ☒No
If Yes, attach the Device Review Sheet.

9. Special Approvals

a. Does this project involve the use of radioisotopes? ☐Yes ☒No
If Yes, attach documentation of approval from the Radiation Safety Division.

b. Does this project include patients with contagious infections (e.g., mumps, measles, chickenpox, TB, meningitis)? ☐Yes ☒No
If Yes, attach documentation of approval from Chairman of the Infection Control Committee of the appropriate facilities.

c. Does this project involve obtaining remnant biopsy or surgical material from the Department of Pathology or any other source? ☐Yes ☒No
If Yes, attach documentation of approval from the entity or individual providing the materials (e.g., the UAB Division of Anatomic Pathology Release of Pathologic Materials).

d. Does this project require obtaining any remnant clinical laboratory specimens, body fluids, or microbiological isolates from the Department of Pathology or any other source? ☐Yes ☒No
If Yes, attach documentation of approval from the entity or individual providing the materials (e.g., the UAB Division of Laboratory Medicine Release of Pathologic Materials).

e. Does this project use stored (existing) specimens from a repository? ☐Yes ☒No

If Yes, attach documentation of approval for use of specimens, and describe how existing specimens are labeled:______

10. Use of Specimens

Does this project involve collecting specimens from participants and storing them for future research? ☒Yes ☐No

If Yes, complete a-h. If no, skip to Item 11

a. How will specimens be obtained, processed, distributed, and stored?

A trained phlebotomist will draw one and a half tablespoons of blood, if participants are breast cancer survivors, phlebotomy will be performed on the arm contra lateral to the cancer (the phlebotomist will verify the appropriate arm prior to each blood draw). A foil covered 10cc EDTA treated vacutainer of blood (two teaspoons), a 9.7cc serum separator vacutainer, and a 2.7cc sodium citrate containing vacutainer will be collected from all participants at baseline, 1- and 2-years follow-up to assess changes over time in biomarkers of successful aging, with the expectation that home gardening will improve these physiologic measures. Blood will be centrifuged within 30 minutes of the draw. Plasma and serum would be drawn-off from each vacutainer and aliquotted into .5 ml cryovials. The samples also will be stored in -80 degree freezers under back up emergency power at Volker Hall G005 and Wallace Tumor Institute 399S until analyzed for plasma alpha carotene, telomerase serum IL-6 and D-dimer. In addition, we will be collecting stool samples to determine the impact of the gardening intervention on the intestinal microbiome. Participants will be asked to use a moistened wipe after they have a bowel movement on the day before their home visit and to then insert the wipe into a plastic bag and record the time and date at which the sample was collected (both the wipe and plastic bag will be mailed to the participant before each of their home visits). The participant will store the sample in their home freezer until the time of the home visit and the sample will be stored in -80 degree freezers under back up emergency power at Volker Hall G005 and Wallace Tumor Institute 399S until analysis of microbiome. Fingernail and toenail clippings as well as saliva samples to assess cortisol levels are collected at the time of home visit and will be stored in -80 degree freezers under back up emergency power at Volker Hall G005 and Wallace Tumor Institute 399S until further analysis of cortisol level (plastic bags and a nail clipper will be mailed to the participant before each of their home visits and they will be encouraged to clip their own nails, but if they are unable, UAB staff will accomplish this during the home visit).

b. How will specimens be labeled (e.g., unique identifier, medical record number, Social Security number, name, date of birth)?

Specimens will be labeled with the study title, a unique study ID number, and the date and time of the blood draw and other samples collection, i.e. stool, saliva, nail clippings.

c. How will clinical data associated with the specimens be collected and stored?

The database will not contain any personal identifying information to link to the subject. The specimens will have a unique identifying code (study ID number). Clinical information will be stored in a separate and protected database that links samples and other information to the subjects.

d. What participant-identifying information will be collected and linked to the specimens?

Demographic, diagnosis, and treatment information will be collected on each participant in the study and will be stored in locked study files or in password protected databases.

e. What steps will be taken to maximize the confidentiality of linked identifiers? For example, procedures could include using a password-protected computer database to link identifiers, with limited personnel knowledgeable of the password, or coded identifiers released without the ability to link to clinical data (also called "stripped" or "anonymized" specimens).

The specimens and related data spreadsheets will contain only the subject ID codes to prevent the ability to link personal identifiers. The database requires a secure password, and access is limited. Only research study staff who have received IRB approval are allowed access to the database.

f. Will specimens be shared with other investigators in the future? ☒Yes ☐No
If Yes, what identifiers, clinical information and demographic information will be shared; or will the specimens be stripped of identifiers (i.e., anonymized)? Also **if yes**, outline your procedure for assuring IRB approval for release and use prior to release of specimens.

Currently, we have no definitive plans to share samples; however, we want to keep that option open. Following is the procedure we envision using should the opportunity arise. We would require that any outside collaborating investigators seek and obtain IRB approval at their institution. Once that is obtained, we would file a Material Transfer Agreement. Upon approval specimens would be shipped (note that our specimens will be bar-coded (with a study ID number), the study and PI name and date); thus no personal identifying information is available. As in our previous studies, if we do release biological samples for further secondary analysis, we will keep all other data at UAB and conduct the statistical analysis ourselves and on the UAB site.

Note. Investigators who receive and/or use these specimens must document approval from the appropriate IRB(s) before the specimens may be released.

g. Will biological samples be stored for future use? ☒Yes ☐No

If Yes, indicate whether they will be used for the disease under study in this protocol or research on other diseases.

Any future studies (if any) would likely be limited to cancer in general.

h. Is genetic testing planned? ☐Yes ☒No

If Yes, describe the planned testing here and see "DNA/Genetic Testing" in the Guidebook for consent requirements.

______

11. Gene Therapy

Does this project involve gene therapy or administering recombinant materials to humans? ☐Yes ☒No

If Yes, submit the Gene Therapy Project Review Panel Report –OR- If this is a vaccine trial that is exempt from the NIH Guidelines For Research Involving Recombinant DNA Molecules, submit the Protocol Oversight Review Form For Clinical Vaccine Trials.

12. HIPAA Privacy and Security

Will the PI or others obtain, review, or make other use of participants' "personal health information" (i.e., information, whether oral or recorded in any form or medium that (a) is created or received by a health care provider and (b) relates to past, present, or future physical or mental health or condition of an individual; or provision of health care; or payment for provision of heath care)? ☒Yes ☐No

If Yes, complete a-e as described.

a. Will the data/information be stored or managed electronically (on a computer)? ☒Yes ☐No

b. Is the principal investigator requesting that the UAB IRB waive patient HIPAA authorization from another institution or entity (e.g., insurance company, collaborating institution). ☐Yes ☒No

If Yes, attach copy of privacy notices from institution/entity, and provide the name of institution/entity:______

c. Indicate which, if any, of the listed entities below would provide information or maintain health information collected for this protocol and/or where health information that been collected will be stored/maintained.

☐ The Kirklin Clinic
☒ University of Alabama Hospital
☐ The Children's Hospital of Alabama
☐ Callahan Eye Foundation Hospital
☐ UAB Highlands
☐ Jefferson County Department of Health
☐ School of Dentistry
☐ School of Health Professions
☐ School of Medicine
☐ School of Nursing
☐ School of Optometry
☐ University of Alabama Health Services Foundation

☒ UAB Health Centers
☐ Viva Health
☐ Ophthalmology Services Foundation
☐ Valley Foundation
☐ Medical West - UAB Health System Affiliate
Health System Information Systems:
☐ HealthQuest
☐ Cerner Millennium (Lab, Radiology, UED, Surgery)
☐ EMMI - Master Member Index
☐ Horizon - IPV (IVR/CDA/CRIS)
☐ CareFlow Net
☐ Eclipsys (PIN)
☒ IMPACT
☐ None—**If None, skip to Item 13.**

d. Indicate which of the listed identifiers would be associated/linked with the protected health information (PHI) used for this protocol.
☒ Names
☒ Geographic subdivisions smaller than a State
☒ Elements of dates (except year) related to an individual
☒ Telephone numbers
☐ Fax numbers
☒ Email addresses
☒ Social security numbers
☐ Medical record numbers
☐ Health plan beneficiary numbers
☐ Account numbers
☐ Certificate/license numbers
☐ Vehicle identifiers and serial numbers
☐ Device identifiers and serial numbers
☐ Biometric identifiers
☐ Web universal resource locators (URLs)
☐ Internet protocol address numbers
☐ Full-face photographic images
☒ Any other unique identifying number—Describe: **Unique study ID**
Note. Codes are not identifying as long as the researcher cannot link the data to an individual
☐ None—**If None, skip to Item 13.**

e. Choose one plan to describe your use of the personal health information:
☐ The data collected meet the specifications for a "limited data set"
—Attach Data Use Agreement or Business Associate Agreement

☒ Research staff will obtain authorization from each patient to use the information
—Attach Patient Authorization form, complete except for patient name and IRB protocol number

☐ PI requests Waiver of Patient Authorization to use the information
—Attach Waiver of Authorization and Informed Consent form

PROPOSED RESEARCH

- The IRB will not accept grant applications and/or sponsor's protocols in lieu of the items as outlined below.
- Do not separate responses from items. Instead, insert your response to each item below the item, keeping the information in the order of this form.
- Number each page of the Human Subjects Protocol (i.e., Page *X* of *Y*).

13. Purpose—in nontechnical, lay language

Summarize the purpose and objectives of this protocol, including any related projects, in one short paragraph.

Based on our three successful gardening interventions that paired cancer survivors with Cooperative Extension Master Gardeners (MG) since 2011, we now propose a randomized controlled trial among a statewide sample of 426 older cancer survivors. The purpose of this larger study is to determine the efficacy of a gardening intervention that would pair older cancer survivors (≥ 65 years of age) with master gardeners. Vegetable gardens will be established at survivors' homes, and a yearlong intervention involving bimonthly contact between survivors and MGs to plan and plant gardens, check plant status, harvest, and rotate plantings will be conducted. We hypothesize that this intervention will significantly improve fruit and vegetable consumption, physical activity, quality of life and physical functioning.

14. Background—in nontechnical, lay language

Summarize in 2-3 paragraphs past experimental and/or clinical findings leading to the formulation of this study. Include any relevant past or current research by the Principal Investigator. For drug and device studies summarize the previous results (i.e., Phase I/II or III studies).

There are roughly 15 million cancer survivors in the US, comprising 4% of the populace. The number of cancer survivors is skyrocketing due to a confluence of the following factors: 1) Americans are aging; 2) cancer is an age-related disease (>60% of cancer survivors are age 65+); and 3) improvements in early detection and treatment have resulted in many common cancers having 5-year cure rates that surpass 90%. Rising numbers of cancer survivors is good news, but over $130 billion annually is needed to address their long-term health and psychosocial needs. Compared to others, cancer survivors are at higher risk for other cancers, CVD, osteoporosis, and diabetes. Accelerated functional decline also is a major problem for cancer survivors, especially those who are older. Baker et al. found that compared to age-matched controls, cancer cases (n=45,494) had significantly lower physical and social functioning, vitality, mental health, and HRQoL ($p<0.001$). Results of others are similar and suggest that cancer survivors face functional decline that threatens their ability to live independently, posing a burden to themselves, their families and the health care system. Thus, cancer survivorship is claimed as a national priority, and there is a call for interventions that target markedly vulnerable subsets.

Prior interventions to improve physical activity (PA) and diet quality (DQ) have proven effective in improving functional status and other health

outcomes in cancer survivors. The 2 largest randomized controlled trials (RCTs) to date aimed at physical function, RENEW (Reach-out to ENhancE Wellness, n=641) and Project LEAD (Leading the way in Exercise And Diet, n=182), were led by Demark-Wahnefried (PI) and tested home-based interventions. Both RCTs resulted in significant improvements in DQ, PA and physical function, with few adverse events and low rates of attrition, but there was little capacity for dissemination once funding ended. Thus, we have sought to develop interventions that build on existing programs and that have a high likelihood of translation into the community. The proposed intervention relies on: (a) the extant infrastructure of the Alabama Cooperative Extension, (b) our collaborative pilot studies with Master Gardeners (MGs), (c) our data that show that gardening consists of low-to-moderate PA that combines aerobic and strength training activities associated with improved health, and (d) new observational data published in 2012 that show that gardeners have significantly better gait speed, balance, and significantly fewer chronic conditions, functional limitations and falls.

MG Programs exist in land grant universities in all 50 United States. Certified MGs complete > 100 hrs. of instruction and community service (CS) and 25 hrs./year of CS to maintain active status. In surveying 184 MGs in AL, we found that 71% were "extremely interested" in mentoring a cancer survivor on vegetable gardening for their CS, and an extra 26% stated that "they were interested and wanted to learn more." Thus, the project is of great interest and builds on an extant infrastructure for sustainability. Ultimately, this intervention could be disseminated to states with 3 growing seasons and adapted to colder weather in those with 2 growing seasons. The intervention also could be adapted for persons with other types of chronic disease in which physical functioning and lifestyle behaviors are key. Finally, this project is significant because the intervention has great potential for sustainability since gardening: (a) involves many activities which prevent satiation common with other forms of exercise; (b) provides a sense of achievement and zest for life that come from nurturing and observing new life and growth; and (c) imparts natural prompts since plants require regular care (watering) and attention (harvesting) and serve as continual and dynamic behavioral cues.

15. Participants (Screening and Selection)

a. How many participants are to be enrolled at UAB? **426 older cancer survivors**
If multi-center study, total number at all centers:______

b. Describe the characteristics of anticipated or planned participants.
Sex: **Male and Female**

Race/Ethnicity: **Our sample will represent the state population for race-ethnicity (27% minority).**

Age: **65 and older**
Health status: **Men and women recently diagnosed with a cancer of favorable prognosis (localized and regional staged female breast, and**

prostate cancers; localized colon & rectum, uterine cervix & corpus, kidney/renal pelvis, non-Hodgkin lymphoma, oral cavity/pharynx, and esophagus cancers; and in situ bladder cancer); and have no medical conditions that would preclude gardening or the consumption of a diet high in fruits and vegetables (e.g., pharmacologic doses of warfarin) – see inclusion/exclusion criteria following page.

Note. *If data from prior studies indicate differences between the genders or among racial/ethnic groups in the proposed research or if there are no data to support or to negate such differences, Phase 3 clinical trials will be required to include sufficient and appropriate entry of gender and racial/ethnic subgroups so that trends detected in the affected subgroups can be analyzed. If ethnic, racial, and gender estimates are not included in the protocol, a clear rationale must be provided for exclusion of this information. If prior evidence indicates that the results will not show gender or racial differences, researchers are not required to use gender or race/ethnicity as selection criteria for study participants. They are, however, encouraged to include these groups. See Section II. Policy of the NIH POLICY AND GUIDELINES ON THE INCLUSION OF WOMEN AND MINORITIES AS SUBJECTS IN CLINICAL RESEARCH – Amended, October, 2001) for further details.*

c. From what population(s) will the participants be derived?

Please note that as in previous studies, like our ENERGY trial (F100325003), we will ascertain cases from the Alabama State Cancer Registry and they will obtain approval from patients' oncology care physicians for us to contact their patients for this study using the letter of invitation provided in our packet of materials (note that in providing us permission to contact their patients, physicians of record also would provide their signature to include in this letter of invitation). In order to identify participants (for recruitment purposes) via the state cancer registry, we will obtain PHI prior to enrollment (see Waiver). We also will ascertain cases directly from the UAB cancer registry and from sites associated with the UAB cancer care network. Additionally, we will post an advertisement material in local newspapers to obtain self-referred participants (see attached), and also will contact individuals who had asked that we keep their names on a wait-list should we embark on another study (Electronic Database for Wait-Listed Patients UAB IRB Protocol Number: X101104003).

Describe your ability to obtain access to the proposed population that will allow recruitment of the necessary number of participants:

As mentioned, cancer registries will provide us with names and addresses of adult men and women (age 65 and older) living in Alabama, who have been diagnosed with loco-regionally staged cancers with good prognoses (≥80% 5-year relative survival) and who have been approved for contact by their oncology care provider. A direct mail approach is the best way to assure that survivors, regardless of county of residence, cancer diagnosis,

age, race, and socio-economic status are all given an equal opportunity to participate. We have used this approach successfully in the past (ENERGY trial) and 91% of oncology care physicians within the area have provided approval for contacting their patients about diet and exercise studies. The mailing will include the letter of invitation (co-signed by the physician of record) and the study screener (see attached materials). For self-referred participants who respond to our advertisements, we will use the study screener to assess eligibility over the phone, and also will follow this procedure when we contact individuals who had asked that we keep their names on a wait-list should we embark on another study (Electronic Database for Wait-Listed Patients UAB IRB Protocol Number: X101104003). Since the activity monitor, stool and nail samples and questionnaires are likely to be completed prior to the in-home baseline visit, it will be necessary to conduct informed consent via telephone. Men and women who are deemed eligible will be telephoned and a phone consent time scheduled (see attached materials for the consent script). Participants will receive a mailed copy of the consent form as well as a postage paid envelope to return the consent. After the telephone consent has been completed and returned, the baseline visit will be scheduled. Roughly 10-12 days prior to the baseline visit, participants will be mailed questionnaires, a programmed accelerometer with instructions for a 7-day collection and a moistened wipe and collection bags for the stool and nail clipping samples. These materials will be collected by study staff during the baseline visit conducted in participants' homes.

Describe the inclusion/exclusion criteria:

We will include individuals who: (1) are diagnosed with a loco-regionally staged cancer associated with an 80% or greater 5-year survival rate (localized and regional staged female breast, and prostate cancers; localized colon & rectum, uterine cervix & corpus, kidney/renal pelvis, non-Hodgkin lymphoma, oral cavity/pharynx, and esophagus cancers; and in situ bladder cancer); (2) reside in the state of Alabama; (3) completed primary curative cancer treatments, i.e., surgery, chemotherapy or radiation therapy; (4) are at least 65 years of age; (5) are at higher risk of functional decline (≥ 2 physical function (PF) limitations as defined by the SF36 PF subscale); (6) currently eat less than 5 servings of fruits and vegetables (F/V)/ day; (7) exercise less than 150 minutes/ week; (8) speak and write in English (some of our scales are not validated in other populations/languages); and (9) are willing to be randomized to either study arm and participate in the follow-up assessments. We will exclude individuals who: (1) are not competent due to mental health or other very serious comorbid conditions (e.g., severe orthopedic conditions or scheduled for a hip or knee replacement with 6 months, paralysis, unstable angina or who have experienced a myocardial infarction, congestive heart failure or pulmonary conditions that require hospitalization or oxygen within 6 months, stroke, degenerative neurological conditions); (2) have any medical condition substantially limiting moderate physical activity; (3) currently taking pharmacologic

doses of warfarin (does not include doses taken to maintain a port); (4) do not reside in a location that can accommodate 4 or more Earthboxes or 1-raised bed (4'x 8'), and that get at least 4 hours of sun a day; (5) do not have running water; (6) have recent experience with vegetable gardening, e.g. planted a vegetable garden within the past year; or (7) have a history of lymphedema flares, axillary node dissection of 10 or more lymph nodes per side.

d. If participants will comprise more than one group or stratification, describe each group (e.g., treatment/intervention, placebo, controls, sham treatment) **and** provide the number of participants anticipated in each group.

Participants will be recruited into 8 cohorts [From Cohort 1 (Start in Year 1) to Cohort 8 (Start in Year 3)] to achieve our accrual goal of 426 participants. Each cohort will be comprised of 52-54 participants. Participants will then be randomized to 1-of-2 study arms: 1) an intervention group that receives an immediate 1-year mentored vegetable gardening intervention in the first year (26-27 participants), or 2) a wait-listed group that receives a delayed 1-year mentored vegetable gardening intervention in the second year (26-27 participants). All participants will be on study for two years. Participants will be block randomized within 2 strata defined by the number of physical function limitations as assessed by the SF36-PFSS (2 vs. ≥3). Participants would be assigned with equal allocation either group A or group B below:

Group A: Participants assigned to this group, would be paired immediately with a Master Gardener from the Cooperative Extension in their county of residence. They also would be given supplies (soil, plants, seeds, and fertilizer) to support either 4 earth boxes (which can be used to garden on balconies, patios or decks) or 1 raised bed garden. These supplies will be provided free of charge. Master Gardeners will make monthly visits to guide participants in setting-up the garden, maintaining it, and replanting it season-to-season. Every month, Master Gardeners also will contact survivors by telephone or email (based on survivor preference) to check-in on garden status and to field questions or problems.

Group B: Participants assigned to this group, would receive the exact same program as Group A (see above), but would receive it AFTER a waiting period of one year. During the wait period, UAB study staff will send participants quarterly holiday postcards reminding them about the study and letting them know that we are looking forward to delivering the program to them in the upcoming year. These postcards will encourage wait-listed participants to contact us if their address or health status changes. These postcards are planned for New Year's, St Patrick's Day, 4th of July and Thanksgiving.

e. Indicate which, if any, of the special populations listed below will be involved in the protocol. Include the Special Populations Review Form (SPRF) if indicated.

☐ Pregnant Women: Attach SPRF—Pregnant Women, Fetuses, Neonates/Nonviable Neonates

☐ Fetuses: Attach SPRF—Pregnant Women, Fetuses, Neonates/Nonviable Neonates

☐ Neonates/Nonviable Neonates: SPRF—Pregnant Women, Fetuses, Neonates/Nonviable Neonates
☐ Prisoners: Attach SPRF—Prisoners
☐ Minors (<18 years old): Attach SPRF—Minors
☒ Employees or students at institution where research conducted
☐ Persons who are temporarily decisionally impaired
☐ Persons who are permanently decisionally impaired (e.g., mentally retarded)
☐ Non-English Speakers

For each box checked, describe why the group is included **and** the additional protections provided to protect the rights and welfare of these participants who are vulnerable to coercion:

Student and employees will not be targeted specifically for recruitment; however, if a potential participant is identified through the cancer registries and is a student or employee, their participation or non-participation will not affect either their employment or academic standing at UAB.

f. List any persons other than those directly involved in the study who will be at risk. If none, enter "None": **None**

g. Describe the process (e.g., recruitment, chart review) that will be used to seek potential participants (e.g., individuals, records, specimens). Research recruitment by non-treating physicians/staff may require completion of Partial Waiver of Authorization for Recruitment/Screening. (See http://main.uab.edu/show.asp?durki=61981.)

Cancer survivors (name and address obtained from the Alabama State Cancer Registry, as well as specific hospital registries associated with UAB or the UAB cancer care network) who live within a 15 mile radius of Master Gardener (MG) volunteers will be mailed a letter of invitation that explains the program, i.e., a 2-year program in which the participant may be randomized to the immediate gardening intervention or the delayed gardening intervention (wait-list control) where they would work with a MG and would be mentored and receive the supplies necessary to establish spring, summer and fall gardens or to a usual care group, as well as to participate in baseline and one- and two-year follow-up assessments. The letter (which would be co-signed by their physician of record) would make it clear that the program was free-of-charge, and that a small incentive of $25 would be provided upon completion of each of the three assessments. The letter would also mention $10 would be provided after completion of each questionnaires with return postage at 6- and 18-months. Letters will include a telephone number and encouragement will be given to call if survivors have questions or concerns, or if they wanted to enroll in the program immediately. A brief questionnaire that collects information on interest in participation and basic eligibility criteria (if interested) or reasons for disinterest (if not interested) would be included within the letter along with a pre-addressed postage-paid envelope for return. Based on a 21% response rate obtained from our previous study, we will mail letters out in 8

batches, first starting with a batch of 265 and then mailing in progressively during a recruitment period of 2½ years until we ascertain our targeted accrual. By using this method we should get a representative and accurate assessment of uptake without putting ourselves into the position of offering the program to more survivors than we have the resources to accommodate.

h. If you will use recruitment materials (e.g., advertisements, flyers, letters) to reach potential participants, attach a copy of each item. If not, identify the source (e.g., databases) from which you will recruit participants.

We will recruit participants primarily from lists provided by the Alabama State Cancer Registry (ASCR) as well as specific sites affiliated with UAB cancer care network. Additionally, we will post an advertisement material in the local newspapers to obtain self-referred participants. We also will contact individuals who had asked that we keep their names on a wait-list should we embark on another study (Electronic Database for Wait-Listed Patients UAB IRB Protocol Number: X101104003). The letter of invitation, eligibility screener and scripts, and the poster are attached.

i. Describe the procedures for screening potential participants.

Participants will be mailed a screener with their letter of invitation. The cancer survivors will be asked to answer the questions on the screener if interested in participating in the study and will return to study staff in the postage paid envelope provided. (See attached screener for specific questions asked). For self-referred participants who respond to our advertisements or those in our wait-list database, we will use the study screener to diagnose eligibility over the phone.

16. Protocol Procedures, Methods, and Duration of the Study—in nontechnical language

a. Describe the procedures for all aspects of your study. Tell us what you are doing.

Assessments:
To measure the impact of the program on the lifestyle behaviors and health of study participants, home visits will be scheduled will all participants (to assess survivor's health and health behaviors at baseline and at the time of consent, and then again 1-year and 2-year later. In addition to home visits, participants will be mailed questionnaires with return postage at 6- and 18-month after study start. Assessments will provide information on specific domains. The assessments are described below and are attached to this application:

<u>Physical Function</u>

<u>SF36 Physical Function subscale (SF36 PFSS)</u>:
This 10-item subscale assesses general physical function and is valid & reliable for use in healthy & chronically-ill adults.

Senior Fitness Test Battery:
Measures physical function in four domains: (1) lower & upper body strength (30-sec chair stand, arm curl); (2) endurance (2-min step test); (3) flexibility (chair sit-&-reach, back scratch); and (4) agility/dynamic balance (8-ft Get Up & Go).

Grip Strength:
Measures participant's functional limitations and disability using a dynamometer.

Usual and rapid gait speed:
Predictive of functional health and mortality

Biomarkers of Physical Function and Healthful Aging
Blood will be drawn in a 10cc EDTA treated vacutainer, a 9.7cc serum separator vacutainer, and a 2.7cc sodium citrate containing vacutainer allowed to clot & centrifuged immediately (portable centrifuges will be used in the field as in our epidemiologic studies). Plasma and serum would be drawn-off from each vacutainer and aliquotted into .5 ml cryovials. The samples also will be stored in -80 degree freezers under back up emergency power at Volker Hall G005 and Wallace Tumor Institute 399S until analyzed for plasma alpha carotene, telomerase, serum IL-6 and D-dimer.

Intestinal Microbiome
We will be collecting a stool sample to determine the impact of the gardening intervention on the intestinal microbiome. Participants will be asked to use a moistened wipe after they have a bowel movement on the day before their home visit and to then insert the wipe into a plastic bag and record the time and date at which the sample was collected (both the wipe and plastic bag will be mailed to the participant before each of their home visits). The participant will store the sample in their home freezer until the time of the home visit and the sample will be stored in in -80 degree freezers under back up emergency power at Volker Hall G005 and Wallace Tumor Institute 399S until analysis of microbiome.

Cortisol Level
We will be collecting toe and finger nail clippings and saliva samples to determine the impact of the gardening intervention on cortisol (a biomarker indicating stress). Participants will be asked to clip their toe and finger nails on the day or before their home visit and to then insert into a plastic bag and record the time and date at which the sample was collected (plastic bags and a nail clipper will be mailed to the participant before each of their home visits). UAB staff will clip the nails of participants who are unable to clip their own nails. Nail clipping samples will be handed to study staff at the time of the home visit. Saliva sample also will be collected at the time of home visit. All specimens will be stored in -80 degree freezers under back up emergency power at Volker

Hall G005 and Wallace Tumor Institute 399S until analysis of cortisol level.

Fruit and Vegetable Dietary Intake
Eating at America's Table Screener (EATS), a10-item questionnaire developed by the NCI will be used to assess fruit and vegetable dietary intake.

Physical Activity

Community Healthy Activities Models Program for Seniors (CHAMPS)
This measure captures physical activities specific to older adults

Accelerometry
PA will be objectively measured via accelerometry (Actigraph, Fort Walton Beach FL). Programmed units will be mailed to participants with instructions for 7 day collection, and then collected by staff during home visits. As with any study measure or particular questionnaire item, while we will encourage participants to complete this procedure, if they refuse, they will still be allowed into the study. This unit was selected given its reliability & longstanding use in large scale trials. Accelerometers will be downloaded & processed using procedures & software supplied by the manufacturer.

Health Related Quality of Life
Health related quality of life will be measured using the 36-item SF36 Health-Related QoL Index (SF36v2r).

Anthropometrics
Height, weight, and waist circumference will be measured during home assessments using a calibrated scale (to nearest 0.1 kg), a portable stadiometer (nearest 0.5 cm), and a non-stretch, tension-controlled tape measure (nearest 0.5 cm), respectively.

Comorbidity
The Older Americans Resources & Services (OARS) Comorbidity Index will be used to assess the number of chronic medical conditions/ symptoms and their functional impact (severity).

Reassurance of Worth
One of 6 subscales of the Revised Social Provision Scale, this measure will be used to assess the psychosocial benefits of gardening. Several gardening studies have reported enhanced self-esteem, increased independence, and increased zest for life.

Mediators
1. Community-Level: Participant-MG dyads will independently assess the participant's local environment for support of vegetable

gardening considering the following factors: 1) availability of garden stores; 2) presence of pests (i.e., insects, deer); 3) neighborhood covenants that impose landscaping restrictions; and 4) sense of belonging with other gardeners in local community.

2. **Interpersonal: We will use the Social Support & Eating Habits (10 items) & Exercise Surveys.**

3. **Individual: This assessment will measure the participant's self-efficacy (survivors' beliefs in their ability to maintain a successful vegetable garden).**

Intentions of Future Gardening
Upon completion of the intervention, participants will be asked whether they intend to continue vegetable gardening and to expand their gardening space.

Demographic & Health-Related Characteristics
Data will be collected on age, race / ethnicity, education, income range, marital status, occupation, and smoking status. Cancer related data will be obtained from both participants (treatment: surgery, radiotherapy, chemotherapy, hormone therapy) and the cancer registry (cancer site / stage, diagnosis year).

Process data
This data (emails, photographs, home visits, telephone calls, etc.) will be collected and used to evaluate adherence to and fidelity of the intervention.

b. What is the probable length of time required for the entire study (i.e., recruitment through data analysis to study closure)?
5 years

c. What is the total amount of time each participant will be involved?
2 years

d. If different phases are involved, what is the duration of each phase in which the participants will be involved? If no phases are involved, enter "not applicable."

Participants will be involved in the study for two years. The tentative timeline for the project is presented in this table:

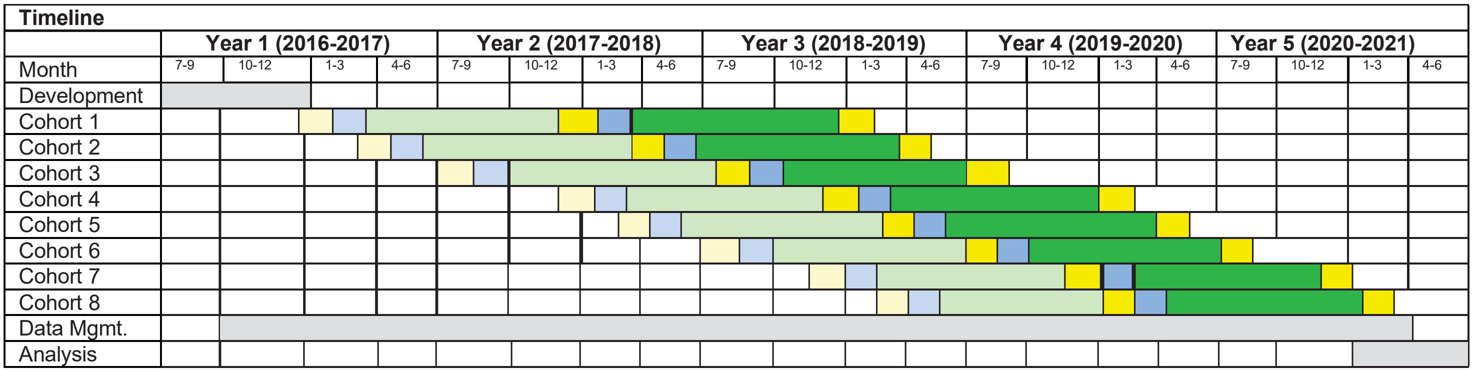

e. List the procedures, the length of time each will take, and the frequency of repetition, and indicate whether each is done solely for research or would already be performed for treatment or diagnostic purposes (routine care) for the population. *Insert additional table rows as needed.*

**Senior Fitness Test Battery and other physical function**			
Procedure	Length of Time Required of Participants	Frequency of Repetition	Research (Res) –OR- Routine Care
Timed 8' Get Up and Go	<1 minute	3 times (baseline, 1-year and 2-year follow-up)	☒Res ☐Routine
Arm Curl	<1 minute	3 times (baseline, 1-year and 2-year follow-up)	☒Res ☐Routine
2-minute Step Test	2 minute	3 times (baseline, 1-year and 2-year follow-up)	☒Res ☐Routine
30 second Chair Stand	30 seconds	3 times (baseline, 1-year and 2-year follow-up)	☒Res ☐Routine
Chair Sit-and-Reach	<1 minute	3 times (baseline, 1-year and 2-year follow-up)	☒Res ☐Routine
Back Scratch	<1 minute	3 times (baseline, 1-year and 2-year follow-up)	☒Res ☐Routine
Grip Strength	<1 minute	3 times (baseline, 1-year and 2-year follow-up)	☒Res ☐Routine
Usual and Rapid Gait Speed	<1 minute	3 times (baseline, 1-year	☒Res ☐Routine

		and 2-year follow-up)	

Physical Activity			
Procedure	Length of Time Required of Participants	Frequency of Repetition	Research (Res) –OR- Routine Care
Accelerometry	7 days	3 times (baseline, 1-year and 2-year follow-up)	☒Res ☐Routine
Community Healthy Activities Models Program for Seniors (CHAMPS)	5 minutes	5 times (baseline, 6 months, 1-year, 18 months, and 2-year follow-up)	☒Res ☐Routine

Biomarkers of Physical Function & Healthful Aging			
Procedure	Length of Time Required of Participants	Frequency of Repetition	Research (Res) –OR- Routine Care
Blood Draw	1 minute	3 times (baseline, 1-year and 2-year follow-up)	☒Res ☐Routine

Intestinal Microbiome			
Procedure	Length of Time Required of Participants	Frequency of Repetition	Research (Res) –OR- Routine Care
Stool Sample	<1 minute	3 times (baseline, 1-year and 2-year follow-up)	☒Res ☐Routine

Cortisol Level			
Procedure	Length of Time Required of Participants	Frequency of Repetition	Research (Res) –OR- Routine Care
Saliva	2 minutes	3 times (baseline, 1-year and 2-year follow-up)	☒Res ☐Routine
Toe and finger nail clippings	2 minutes	3 times (baseline, 1-year and 2-year follow-up)	☒Res ☐Routine

Anthropometrics

Procedure	Length of Time Required of Participants	Frequency of Repetition	Research (Res) –OR- Routine Care
Height	<1 minute	Once at baseline	☒Res ☐Routine
Weight & waist circumference	2 minutes	3 times (baseline, 1-year and 2-year follow-up)	☒Res ☐Routine

Comorbidity			
Procedure	Length of Time Required of Participants	Frequency of Repetition	Research (Res) –OR- Routine Care
OARS	3 minutes	3 times (baseline, 1-year and 2-year follow-up)	☒Res ☐Routine

Fruit and Vegetable Dietary Intake			
Procedure	Length of Time Required of Participants	Frequency of Repetition	Research (Res) –OR- Routine Care
Eating at America's Table Screener (EATS)	3 minutes	5 times (baseline, 6 months, 1-year, 18 months, and 2-year follow-up)	☒Res ☐Routine

Mediators			
Procedure	Length of Time Required of Participants	Frequency of Repetition	Research (Res) –OR- Routine Care
Community Support	3 minutes	3 times (baseline, 1-year and 2-year follow-up)	☒Res ☐Routine
Social Support & Eating Habits	5 minutes	3 times (baseline, 1-year and 2-year follow-up)	☒Res ☐Routine
Self-efficacy	3 minutes	3 times (baseline, 1-year and 2-year follow-up)	☒Res ☐Routine

Surveys			
Procedure	Length of Time Required of Participants	Frequency of Repetition	Research (Res) –OR- Routine Care

The Social Provisions Scale	5 minutes	3 times (baseline, 1-year and 2-year follow-up)	☒Res ☐Routine
Health Related Quality of Life (SF36v2r) SF36 Physical Function subscale (SF36 PFSS)	5 minutes	3 times (baseline, 1-year and 2-year follow-up)	☒Res ☐Routine
Intention of Future Gardening	<1 minute	Once at 1-year or 2-year follow-up	☒Res ☐Routine
Demographic and Health-Related Characteristics	<1 minutes	Once at baseline	☒Res ☐Routine
Process Data- i.e. emails, photographs, home visits, telephone calls	30 minutes	Monthly	☒Res ☐Routine

f. Will an interview script or questionnaire be used? ☒Yes ☐No
If Yes, attach a copy.

g. Will participants incur any costs as a result of their participation? ☐Yes ☒No
If Yes, describe the reason for and amount of each foreseeable cost.

______

h. Will participants be compensated? ☒Yes ☐No
If Yes, complete i-v:
i. Type: (e.g., cash, check, gift card, merchandise): **Cash and garden supplies**
ii. Amount or Value: **$25 for study visits, $10 for questionnaire completion, and approx. $500 in gardening supplies**
iii. Method (e.g., mail, at visit): **at visit**
iv. Timing of Payments: (e.g., every visit, each month):

A staff member will make a home visit to each of the 426 participants enrolled in each cohort shortly after consent and again 1-year and 2-years later. Participants will be compensated with the completion of each assessment (i.e. $25 per visit). In addition, participants will be compensated with the completion of postage paid questionnaires at 6- and 18-months (i.e. $10 per mailing). The survivor in the immediate intervention group will receive approximately $500 in gardening supplies over the course of the intervention period. The survivor in the delayed intervention group will receive gardening supplies 1-year later of the study start.

v. Maximum Amount of Payments per Participant: **$95**

17. Describe the potential benefits of the research.

The benefit to society is reasonably high since the proposed intervention may help improve lifestyle behaviors that could potentially impact progressive disease and co-morbidities in cancer survivors. Thus, this project serves many purposes: (1) To explore the feasibility and acceptability of a mentored vegetable gardening intervention. This aim will be accomplished by collecting detailed process data, which will allow the assessment of accrual, retention, adherence/fidelity, and adverse events; (2) To quantify (mean change scores and precision estimates) and compare between-arm differences in physical function as measured by the Short Form 36 Physical Function Subscale (SF36-PFSS); (3) To explore participant factors associated with program efficacy, e.g., gender, and comorbidity; and (4) To obtain means and precision estimates and explore between-arm differences on pre-post changes in secondary endpoints: (a) physical performance tests (Short Physical Performance Battery (SPPB) & Senior Fitness Tests), (b) biomarkers of physical function and healthful aging (e.g. IL-6, D-dimer, & telomerase), (c) F&V intake and diet quality (DQ), (d) accelerometer measured PA, (e) BMI, (f) health-related quality of life (HRQoL), and (g) reassurance of worth.

18. Risks

a. List the known risks—physical, psychological, social, economic, and/or legal—that participants may encounter as a result of procedures required in this protocol. Do not list risks resulting from standard-of-care procedures. *Note. Risks included in this protocol document should be included in the written consent document.*

1). Potential risks include psychological discomfort associated with the survey instruments used for data collection.

2). Subjects in the study may be inconvenienced by scheduled visits by the master gardener or by the paperwork or energies needed to participate in other forms of assessment, e.g., waist circumference.

3). Blood collection involves some risk, as the needle can cause some discomfort and bruising and fainting may result.

4). There is a risk that research data could become linked with an individual's identity, and become available to non-research personnel.

5). Subjects may be inconvenienced or embarrassed providing the stool sample.

b. Estimate the frequency, severity, and reversibility of each risk listed.

1) The survey instruments that will be employed in this study, e.g., mainstream and standardized health related quality of life measures, as well as those used to assess diet and physical activity contain few (if any) psychologically sensitive questions.

2) We have worked diligently to reduce participant burden by employing valid survey scales that are brief. Times for each of the assessments, as

well as for interacting with master gardeners will be scheduled at the patient's convenience, thus we will work around their schedule in order to deliver the intervention and assess its potential impact.

3) Prior to the blood draw, the phlebotomist will verify the side of the previous cancer. Blood will be drawn on the side contra lateral to the affected side. Butterfly needles will be used to perform phlebotomy and a trained phlebotomist will draw blood, thus reducing discomfort and bruising. Furthermore, we have worked to minimize the number of draws to 3 over the course of the 2-year study. This decision is based primarily on previous studies that indicate that blood levels of nutrients are seasonally-influenced, thus the optimal collection periods are 1-year and 2-year from baseline. This also results in fewer draws.

4) The main potential adverse effect is breach of confidentiality regarding health status information reported by a subject. To minimize this potential risk, all data will be stored without personal identifying information and all information will be stored in a password protected file that is only accessible by trained investigators and study staff. Although master gardeners will not collect any information, we will provide training regarding maintenance of confidentiality.

5.) Participants will receive the stool and nail clipping sample collection materials (special wipe and zip lock baggie) prior to their baseline visit so that they can collect these samples in the privacy of their own homes. This should minimize participant embarrassment and inconvenience.

c. Is this a therapeutic study or intervention? ☐Yes ☒No

If Yes, complete the following items:

i. Describe the standard of care in the setting where the research will be conducted:______

ii. Describe any other alternative treatments or interventions:______

iii. Describe any withholding of, delay in, or washout period for standard of care or alternative treatment that participants may be currently using:______

d. Do you foresee that participants might need additional medical or psychological resources as a result of the research procedures/interventions? ☒Yes ☐No

If Yes, describe the provisions that have been made to make these resources available.

Although none of our questionnaires formally assesses at risk conditions, such as depression, there are some items on the SF36 that are aimed at assessing overall physical and mental health and other surveys that assess whether or not individuals have a social network on which they can seek support. If incidental findings are uncovered on this study, such as depression, concerns will be brought to the attention of Dr. De Los Santos, a radiation oncologist who is well familiar with delivering care to breast cancer patients, and appropriate resources will be sought if needed.

e. Do the benefits or knowledge to be gained outweigh the risks to participants?
☒Yes ☐No

If No, provide justification for performing the research:______

19. Precautions/Minimization of Risks

a. Describe precautions that will be taken to avoid risks and the means for monitoring to detect risks.

The risks associated with the proposed study are minimal since moderate amounts of physical activity and a healthy diet generally promote good health, not endanger it. However, there are participants for whom such a tact is contraindicated. Our eligibility criteria are specifically established to exclude individuals for whom unsupervised physical activity and home gardening is not appropriate. We also will screen subjects directly using a mailed screener. Thus, using this multi-gated approach, we should be able to effectively screen-out any individuals for whom this intervention is contraindicated. We also will provide articles on gardening safety and health, e.g., protecting knees and back, sun protection, etc. in the study notebook that is delivered to participants prior to starting the intervention (the study notebook is an information source on gardening, health precautions and vegetable preparation).

A private Facebook group will be created to allow interested cancer survivors and Master Gardeners enrolled in the study to obtain and/or share gardening related information, such as the status of their garden (e.g., pictures, what works well, what isn't working), problems they've encountered, solutions to problems, and different ways (e.g., recipes) to enjoy the produce from the garden. Participation in this forum is optional; the opportunity to interact with their fellow study participant-gardeners is being offered per the suggestion of the mini-pilot study participants. UAB study staff and Master Gardeners will not be posting pictures of participants or their gardens to this Facebook page in order to maintain confidentiality. Participants may choose to post pictures themselves. The constructed Facebook page also will include a listing of warning signs with instructions should injury or other health problems occur. A phone number will be provided in order to communicate these with study staff.

All health occurrences will be recorded and regularly reviewed by the study staff. Any serious adverse events will be immediately communicated to Dr. Demark-Wahnefried, who will confer with her co-Investigators (especially clinician-scientist, Jennifer De Los Santos, MD) – a procedure that has worked successfully in the past. All data will be used for research purposes and will be protected and kept strictly confidential. All patients will be asked if and when they suffered adverse events during bi-monthly telephone contact with study staff and will be encouraged to report an event at any time during follow-up. Thus, at any given time, patients will have varying follow-up times. In practice, study participants will be encouraged to call-in on a toll-free number to report any adverse events that would preclude their participation in the study. Data from

these phone calls will be included in the analyses along with data generated by the follow-up surveys (checking for any duplicates and cleaning the data as necessary). Within each arm, we assume independence of events.

If study involves drugs or devices skip Items 19.b. and 19.c., go to Item 20, and complete the <u>Drug</u> or <u>Device</u> Review Sheet, as applicable.

b. If hazards to an individual participant occur, describe (i) the criteria that will be used to decide whether that participant should be removed from the study; (ii) the procedure for removing such participants when necessary to protect their rights and welfare; and (iii) any special procedures, precautions, or follow-up that will be used to ensure the safety of other currently enrolled participants.

The project manager will record all reported events in the adverse event log (including the subject's name, date, and event description). The project manager will inform the principal investigator, Wendy Demark-Wahnefried, PhD, immediately of any unanticipated study deaths or serious events that potentially jeopardize participation in the study. Dr. Demark-Wahnefried will consult with Dr. De Los Santos on the action that should be taken. This communication will occur within 24 hours for an unanticipated study death, and within 5 business days for an unanticipated serious event. If deemed necessary, participants will be removed from the study to ensure their safety. A serious event is defined as "any event or condition that is life threatening, results in overnight hospitalization, cancer or a physical or cardiac event serious enough to require medical attention". A brief listing follows:

- **Fatal**
- **Life threatening**
- **Permanently disabling**
- **Required or prolonged (overnight) hospitalization (Admission—not ER visit)**
- **Overdose**
- **Significant hazard to patient**

c. If hazards occur that might make the risks of participation outweigh the benefits for all participants, describe (i) the criteria that will be used to stop or end the entire study and (ii) any special procedures, precautions, or follow-up that will be used to ensure the safety of currently enrolled participants.

If multiple serious adverse events occur, Dr. Demark-Wahnefried will consult with Dr. De Los Santos on the action that should be taken in terms of continuing the study. If necessary, participants will be informed of the adverse events (via mail or phone) and given the option of continuing the study.

20. Informed Consent

a. Do you plan to obtain informed consent for this protocol? ☒Yes ☐No

If Yes, complete the items below.
If No, complete and include the Waiver of Informed Consent or Waiver of Authorization and Informed Consent, as applicable.

b. Do you plan to document informed consent for this protocol? ☒Yes ☐No
If Yes, complete the items below.
If No, complete the items below **and** include the Waiver of Informed Consent Documentation.

c. How will consent be obtained?

For survivors whom indicate interest, we will initially explain the study over the telephone, assess eligibility and then schedule them for a phone consent appointment. A copy of the consent form will be mailed to the survivor and informed consent will be conducted over the phone at the scheduled time. This will be necessary since the activity monitor willbe worn and the stool and nail clipping sample collected prior to the baseline visit. Since the activity monitors cost approximately $350 each, it will be cost-effective to collect the device at the home visit to ensure that the equipment returns to the lab. Further, the stool sample should be taken after the first bowel movement on the day before the baseline assessment. The toe nail and finger nail clipping samples should be taken before the baseline assessment. Informed consent should be conducted prior to any study measures, thus a phone consent would serve as the most logical procedure to enable these study measures to be conducted prior to the baseline visit. The survivor will be provided with a postage paid envelope to return the consent form to study staff. Once the consent form has been received, the baseline appointment will be scheduled and survivors will be informed that they will receive a copy of the signed consent form for their record, as well as a packet with the activity monitor, stool and nail clipping samples collection kit, and study questionnaires as well as instructions for each. At the time of the baseline appointment, the study would be re-reviewed and further questions answered. The activity monitor, stool and nail clippings samples, and questionnaires will be collected, and the in-home baseline visit will commence.

d. Who will conduct the consent interview? **Project manager, Post-docs, Pre-docs, or Dr. Demark-Wahnefried**

e. Who are the persons who will provide consent or permission? **Older cancer survivor participants**

f. What steps will be taken to minimize the possibility of coercion or undue influence?

Written consent will be obtained during the phone consent appointment which will be scheduled prior to sending study measures (described above) or scheduling the baseline visit. The participant will have time to review the consent form thoroughly prior to the phone consent appointment. Participants will be given the opportunity to ask questions

about the study during the phone consent. Respect for each participant as an autonomous agent will be upheld.

g. What language will the prospective participant or the legally authorized representative understand? **English**

h. What language will be used to obtain consent? **English**

i. If any potential participants will be, or will have been, in a stressful, painful, or drugged condition before or during the consent process, describe the precautions proposed to overcome the effect of the condition on the consent process. If not, enter "no such effect."
No such effect

j. If any project-specific instruments will be used in the consenting process, such as flip charts or videos, describe the instrument(s) here, and provide a copy of each. If not, enter "not used."
Not used

k. How long will participants have between the time they are told about the study and the time they must decide whether to enroll? If not 24 hours or more, describe the proposed time interval and why the 24-hour minimum is neither feasible nor practical. **More than a 24 hour lag time will be employed.**

21. Procedures to Protect Privacy

Describe the provisions included in the research to protect the privacy interests of participants (e.g., others will not overhear your conversation with potential participants, individuals will not be publicly identified or embarrassed).

The data will be published in research and scientific journals without reference to identities of the research volunteers. Research data and samples may be shared with other collaborators at UAB or at other research institutions. In this latter instance, the data and/or sample results will be coded so that there is no reference to identities of the research volunteers; the collaborators will not have any access to identity information.

22. Procedures to Maintain Confidentiality

a. Describe the manner and method for storing research data and maintaining confidentiality. If data will be stored electronically anywhere other than a server maintained centrally by UAB, identify the departmental and all computer systems used to store protocol-related data, and describe how access to that data will be limited to those with a need to know.

All electronic files containing personal identifiers will be stored only within password protected files on the UAB file servers (located behind ample firewalls). Information transferred to the server (for backup purposes) will be done via secure ftp. Files may be transferred to other computers via the Internet. When this is done, the files will be protected through a method that encrypts their contents during transfer and storage. The on-site file servers are physically accessible only to network support specialists (locked rooms). The on-site file server will be electronically accessible only to study staff through user/password

protection. Non-electronic files (such as paper surveys, consent forms, authorization forms from physicians who approve patient contact, etc.) will be kept in a locked file cabinet, located in a locked room. Access to this room is limited to research staff only. In addition, all research staff are HIPAA certified and have completed and are current with regard to IRB training. Data sets provided as a function of the NIH Data Sharing Requirement will be stripped of identifying information according to HIPAA policy.

b. Will any information derived from this study be given to any person, including the subject, or any group, including coordinating centers and sponsors? ☐Yes ☐No
If Yes, complete i-iii.

i. To whom will the information be given?

The information will be given to research & scientific journals, collaborators at UAB/and other research institutions.

ii. What is the nature of the information? **Research data, samples, and results**

iii. How will the information be identified, coded, etc.?

The data will be published in research and scientific journals without reference to identities of the research volunteers. Research data and samples may be shared with other collaborators at UAB or other research institutions. In this latter instance, the data/and or samples will be coded so that there will be no reference to identities of the research volunteers, the collaborators will not have access to identity information. PHI will be stored separately from collected data.

23. Additional Information

In the space below, provide any additional information that you believe may help the IRB review the proposed research, or enter "None."

None
