# Supplementary material for: Vegetable Gardening and Health Outcomes in Older Cancer Survivors: A Randomized Clinical Trial
Source: JAMA Netw Open. 2024 Jun 20;7(6):e2417122. doi: 10.1001/jamanetworkopen.2024.17122 (PMC11190797; doi:10.1001/jamanetworkopen.2024.17122)
Supplement: Supplement 2. — Data Sharing Statement [file jamanetwopen-e2417122-s002.pdf]

# Data Sharing Statement

Demark-Wahnefried. Vegetable Gardening and Health Outcomes in Older Cancer Survivors. *JAMA Netw Open*. Published June 20, 2024. doi:10.1001/jamanetworkopen.2024.17122

**Data**
**Data available:** Yes
**Data types:** Other (please specify)
**Additional Information:** Deidentified data will be made available from Dr. Demark-Wahnefried upon reasonable request. Data to be provided will be group-level data unless participant-level data can be released without revealing an individual's identity.
**How to access data:** Please contact Dr. Demark-Wahnefried at demark@uab.edu
**When available:** With publication

**Supporting Documents**
**Document types:** None

**Additional Information**
**Who can access the data:** researchers whose proposed use of the data has been approved
**Types of analyses:** for a specified purpose that will not compromise the privacy of research participants
**Mechanisms of data availability:** with a signed data access agreement
